# Split NeissLock with Spy-Acceleration Arms Mammalian Proteins for Anhydride-Mediated Cell Ligation

**DOI:** 10.1021/acschembio.5c00515

**Published:** 2025-09-15

**Authors:** Sheryl Y. T. Lim, Anthony H. Keeble, Mark R. Howarth

**Affiliations:** † Department of Biochemistry, University of Oxford, South Parks Road, Oxford OX1 3QU, U.K.; ‡ Department of Pharmacology, 2152 University of Cambridge, Tennis Court Road, Cambridge CB2 1PD, U.K.

## Abstract

Reactive functional groups may be incorporated into proteins or may emerge from natural amino acids in exceptional architectures. Anhydride formation is triggered by calcium in the self-processing module (SPM) of *Neisseria meningitidis* FrpC, which we previously engineered for “NeissLock” ligation to an unmodified target protein. Here, we explored bacterial diversity, discovering a related module with ultrafast anhydride formation. We dissected this swift SPM to generate a split NeissLock system, providing a second layer of control of anhydride generation: first mixing N- and C-terminal NeissLock moieties and second adding millimolar amounts of calcium. Split NeissLock generated a minimal fusion tag, permitting binder expression in mammalian cells with complex post-translational modifications and avoiding self-cleavage while transiting the calcium-rich secretory pathway. Employing spontaneous amidation between SpyTag003 and SpyCatcher003, we dramatically accelerated split NeissLock reconstitution, allowing a rapid high-yield reaction to naturally occurring targets. We established a specific covalent reaction to endogenous Epidermal Growth Factor Receptor using split NeissLock via Transforming Growth Factor-α secreted from mammalian cells. Modular ligation was demonstrated on living cells through site-specific coupling of the clot-busting enzyme tissue plasminogen activator or a computationally designed cytokine. Split NeissLock provides a modular architecture to generate highly reactive functionality, with inducibility and simple genetic encoding for enhanced cellular modification.

## Graphical abstract

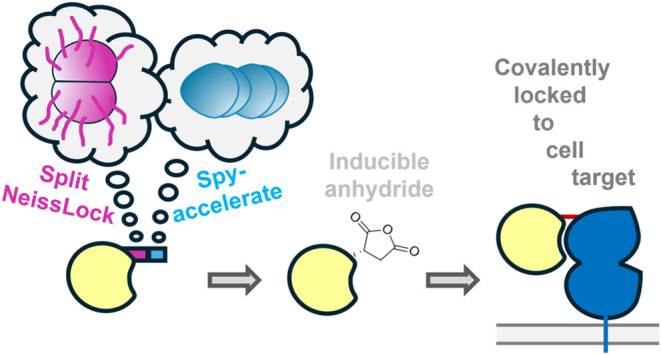

## Introduction

Covalent coupling brings new possibilities for robust and long-lasting assemblies, useful for biotransformation,^1^ diagnostics,^2^ vaccines,^3^ and cell therapies.^4,5^ Highly reactive electrophiles such as acid anhydrides and *N*-hydroxysuccinimides are regularly used for coupling to proteins, including for fluorescent labeling and proteomics.^6,7^ Such classic reactants produce broad, uncontrolled reaction.^6^ For covalent coupling to untagged endogenous proteins with more specificity, electrophiles of lower reactivity, such as acrylamide,^8,9^ chloroacetyl,^10^ or Sulfur­(VI) Fluoride Exchange (SuFEx) probes, are more commonly used.^11^ Employing these weak electrophiles to direct protein–protein ligation may be limited by the cost and complexity of attaching these electrophiles through a separate reaction (e.g., coupling through cysteine)^8,9^ or through noncanonical amino acid mutagenesis.^12–14^ Hence, we have been exploring ways to incorporate electrophiles into proteins using only the standard 20 amino acids.^15,16^ *Neisseria meningitidis* FrpC contains a self-processing module (SPM) that, upon binding calcium, cleaves at the aspartate-proline (D-P) peptide bond, releasing SPM to reveal an aspartic anhydride (Figure 1A).^15,16^ Aspartic anhydride is a highly reactive electrophile, susceptible to rapid reaction with water.^6^ Developing control of this anhydride’s reactivity in diverse contexts would create new opportunities for molecular engineering.

**Figure 1:**
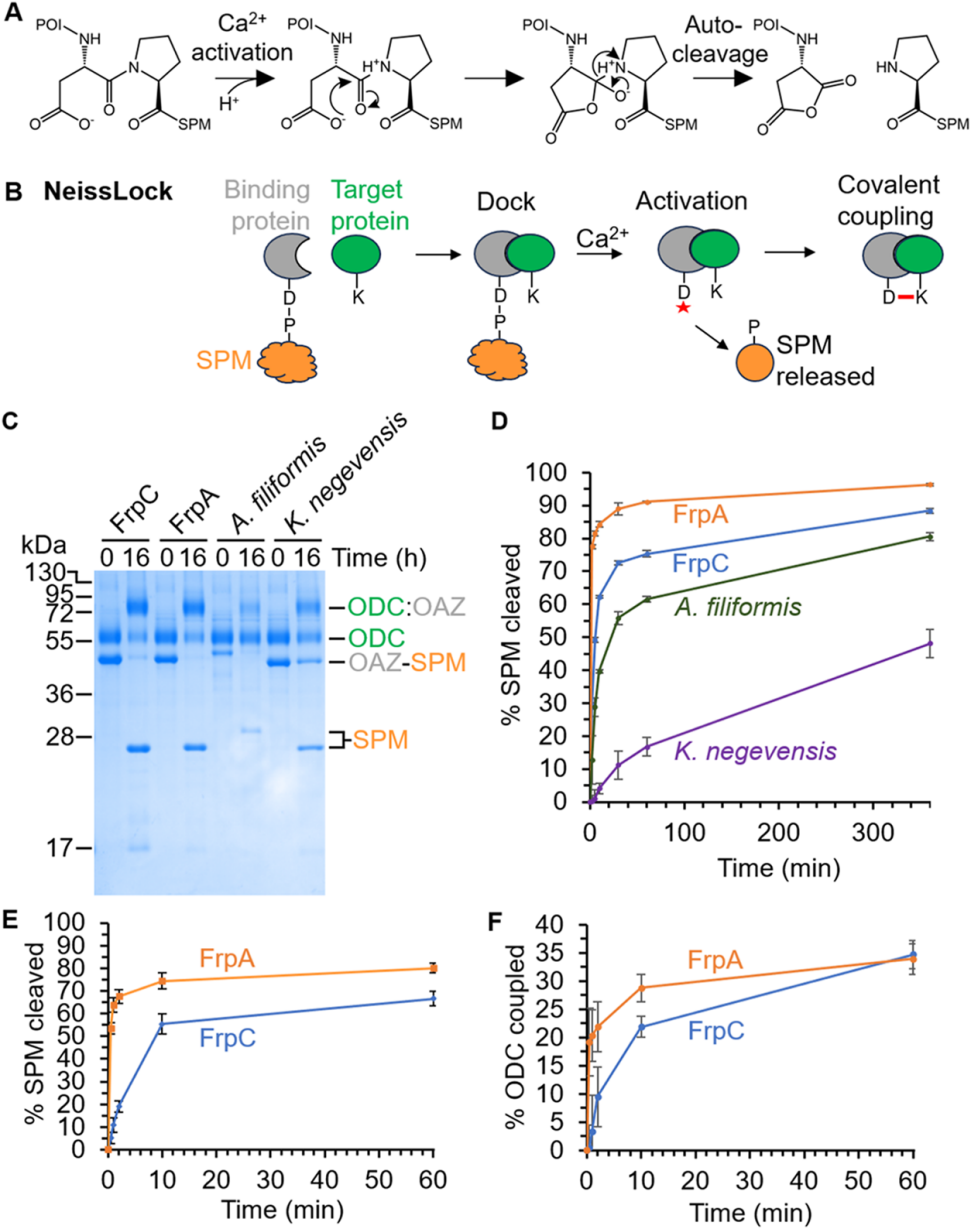
Identifying an ultrafast self-processing module (SPM). (A) SPM undergoes autoproteolysis at Asp-Pro, generating an anhydride. POI is the protein of interest. (B) Schematic of NeissLock. A binding protein genetically fused to SPM docks with a target protein. Upon adding calcium, an anhydride (marked by the red star) is generated on the binding protein, releasing SPM, and enabling covalent coupling to a nucleophile (e.g., lysine, K) on the target. The red line represents an isopeptide bond. (C) Reactivity of SPM homologues. Incubation of different versions of 5 μM OAZ-SPM with 5 μM ODC for 0 or 16 h was done with 10 mM calcium at 37 °C, before SDS–PAGE/Coomassie analysis. A colon indicates covalent coupling. (D) Time course for SPM cleavage. 5 μM OAZ-SPM was mixed with 5 μM ODC for varying times with 10 mM calcium at 37 °C, before SDS–PAGE/Coomassie. (E) SPM cleavage rate for FrpA and FrpC with 10 μM of each partner, after adding 1 mM calcium for the indicated time at 25 °C. (F) Coupling rate was tested as in (E). Plots show mean ± 1 s.d., *n* = 3.

We previously redirected anhydrides for what we termed NeissLock coupling^17^ (Figure 1B), taking advantage of the feature that any protein can be N-terminal to SPM.^16^ In NeissLock, a binding protein that interacts noncovalently with a target is fused with SPM (Figure 1B). The two components are then mixed to form a noncovalent complex, before cleavage at aspartate-proline is initiated with calcium. The resulting anhydride can then react with nearby nucleophiles on the target protein, creating an irreversible covalent complex.^17^ Here, we enhance the NeissLock system by identifying a faster SPM. We then split the SPM so that NeissLock may be performed on proteins expressed in mammalian cells. Through the use of SpyTag/SpyCatcher, we accelerate the reconstitution of the split SPM system. Then, we demonstrate the application of this Spy-accelerated split SPM for covalent ligation of therapeutic proteins to the unmodified Epidermal Growth Factor Receptor (EGFR) at the surface of living cells.

## Results

### Identification of a Faster Reacting SPM Homologue

To advance NeissLock chemistry, here our first step was to explore whether other bacterial systems could give superior inducible formation of anhydrides. We bioinformatically identified a panel of SPMs with varying divergence from *N. meningitidis* FrpC (Figure S1). FrpA SPM from *N. meningitidis* shows a 98% amino acid sequence identity to FrpC SPM. SPM of the hemolysin-type calcium-binding protein-related domain-containing protein from *Alysiella filiformis* shows 71% amino acid sequence identity to FrpC. *A. filiformis* is a nonpathogenic bacterium that infects pigs.^18^ SPM of the bifunctional hemolysin/adenylate cyclase precursor from *Kingella negevensis* shows 60% amino acid sequence identity to FrpC. *K. negevensis* can be found in the throat of children.^19^

As a model for NeissLock coupling, we employed the noncovalent interaction between ornithine decarboxylase (ODC) and ornithine decarboxylase antizyme (OAZ).^17^ After calcium activation of NeissLock coupling, previous mass spectrometry (MS) analysis identified K92 as the primary cross-linking site on ODC, with additional cross-linking to other ε-amines proximal to the C-terminus of OAZ including K121 and K74.^17^ OAZ was genetically fused to the SPM from different species. Each version was efficiently expressed solubly in *Escherichia coli*. All homologues underwent successful calcium-induced cleavage, as well as reaction to the ODC (Figure 1C). FrpA SPM was ultrafast, with 91 ± 0.2% cleaved after 1 h (Figure 1D, mean ± 1 s.d., *n* = 3). The few differences between SPMs of FrpA and FrpC (Figure S2A) have a major effect on the rate. We compared the time course with suboptimal conditions of temperature (25 °C) and calcium (1 mM) (Figures S2B and 1E/F). Cleavage and ligation were also substantially faster for FrpA under these conditions. Nearly 70% FrpA SPM was cleaved in 5 min, while FrpC SPM required 60 min or longer to reach the same cleavage extent. Hence, we utilized the ultrafast FrpA SPM for subsequent engineering. Following calcium addition, the two OAZ species of differing mobility on SDS-PAGE (Figure S2B) correspond to a linear species from hydrolysis of the anhydride and a cyclized species from intramolecular reaction of the anhydride with a residue on OAZ itself, as previously validated by MS.^17^ There is also potential for reaction of the anhydride with the cleaved SPM, but this side-reaction is likely to be less important since the cleaved SPM will be free to diffuse away from the OAZ-anhydride. It is unclear how frequently the SPM side-reaction with the anhydride occurs, since the product will have the same molecular weight as any OAZ-SPM that fails to be activated.

### Engineering a Split SPM to Enable NeissLock Coupling with Mammalian Proteins

It is important for NeissLock to be compatible with binders expressed in the mammalian secretory pathway, since many proteins cannot be functionally expressed in bacteria because of their complex multidomain topology or obligate post-translational modification (e.g., N-linked glycosylation).^20^ However, we foresaw that the millimolar calcium within the mammalian endoplasmic reticulum during secretion^21^ would likely drive precleavage of SPM.^17^ Indeed, when we purified superfolder green fluorescent protein (sfGFP) genetically fused to FrpA SPM, following secretion from human-derived Expi293F cells, a substantial fraction was already cleaved (Figure S3). Aiming to overcome this challenge, we devised a split protein approach, to allow efficient gating of protein function.^22,23^ We designed split FrpA SPM so that NeissLock binding proteins could be expressed with a small inactive N-terminal fragment of SPM (SPM_N_) (Figure 2A). Only upon mixing with a C-terminal fragment of SPM (SPM_C_) should complete SPM be reconstituted, priming calcium-inducible anhydride generation. We initially split between residues 315 and 316 of FrpA SPM, to give an 18-residue N-terminal fragment, to avoid disrupting the central secondary structure (Figure 2B). The N-terminal portion comprised residues 298 to 315, with the C-terminal portion comprising the rest of the SPM. Indeed, we found calcium-induced cleavage and ligation only upon mixing the two fragments (Figure 2C).

**Figure 2:**
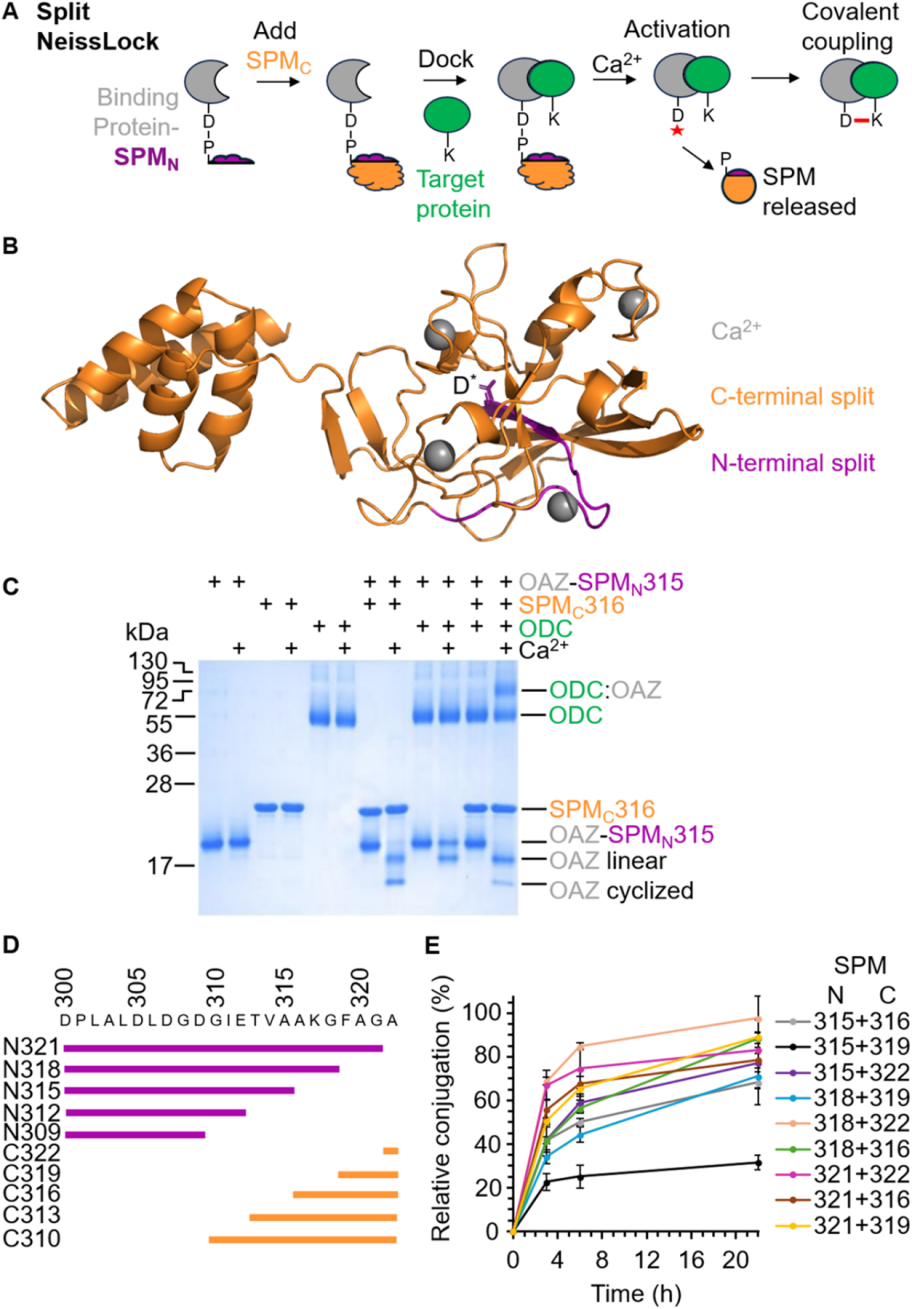
Engineering split NeissLock coupling. (A) Schematic of the split NeissLock. A binding protein genetically fused to the N-terminal fragment of SPM reconstitutes with SPM’s C-terminal fragment, before binding the target protein. Calcium activates anhydride generation, promoting ligation to the target. (B) AlphaFold 3 model of FrpA SPM, color-coded for regions for initial splitting into N-terminal (mauve, residues 300 to 315) and C-terminal (orange, residues 316 to 543) fragments. The reactive aspartate (D*) is shown in a stick format. Ca^2+^ ions are shown as gray spheres. (C) Split NeissLock allows covalent ligation. OAZ-SPM_N_315 and SPM_C_316 each at 10 μM were incubated ± 10 μM ODC ± calcium at 37 °C for 16 h, before SDS–PAGE/Coomassie. (D) Schematic of the different tested SPM_N_ and SPM_C_ fragments. (E) Time course for ligation using SPM_N_ and SPM_C_ fragments. OAZ-SPM_N_ was premixed with SPM_C_, before incubating with ODC (each protein at 5 μM) along with calcium for the indicated times at 37 °C. Reaction was analyzed by SDS-PAGE/Coomassie (mean ± 1 s.d., *n* = 3).

To optimize reconstitution, we varied split positions, and constructs are named after their terminal residue (Figure 2D). Reconstitution was precarious since incubation of SPM_N_309 with SPM_C_310 or SPM_N_312 with SPM_C_313 gave no coupling (Figure S4). However, SPM_N_318 and SPM_C_322 gave excellent reactivity, almost twice that of the original SPM_N_315/SPM_C_316 (Figure 2E). The location of 318 and 322 within a loop of SPM (Figure 2B) is consistent with studies that splitting within loops is best tolerated.^22,23^ Surprisingly, residues 319–321 are absent from the fastest pair. Hereafter, all experiments were performed with SPM_N_318 (N-terminal 298–318; 21 residues) and SPM_C_322 (C-terminal 322–543; 222 residues).

### Spy Ligation Accelerates Split FrpA Cleavage

Despite optimizing split FrpA, reactions took hours (Figure 2E) that would take minutes for full-length SPM (Figure 1F). We hypothesized that inefficient reassembly was limiting cleavage. The peptide SpyTag003 and its protein partner SpyCatcher003 react through a spontaneous isopeptide bond at rates approaching the diffusion limit,^24^ thus having the potential to anchor SPM_N_ to SPM_C_ and facilitate reaction (Figure 3A). SpyTag003 was fused to SPM_N_’s C-terminus, both to minimize the size of the fusion to the binding protein and to minimize the scar left in the covalent complex between the binding protein and the target (Figure 3A). SpyCatcher003 was genetically fused to SPM_C_’s C-terminus through a short glycine/serine-containing spacer. When we modeled the structure of the complex between SPM_N_-SpyTag003 and SPM_C_-SpyCatcher003, AlphaFold 3 predicted the reconstitution of both the SPM_N_/SPM_C_ and SpyTag003/SpyCatcher003 moieties (Figure S5). We validated by MS the spontaneous isopeptide bond formation between the OAZ-SPM_N_-SpyTag003 and SPM_C_-SpyCatcher003 (Figure 3B).

**Figure 3:**
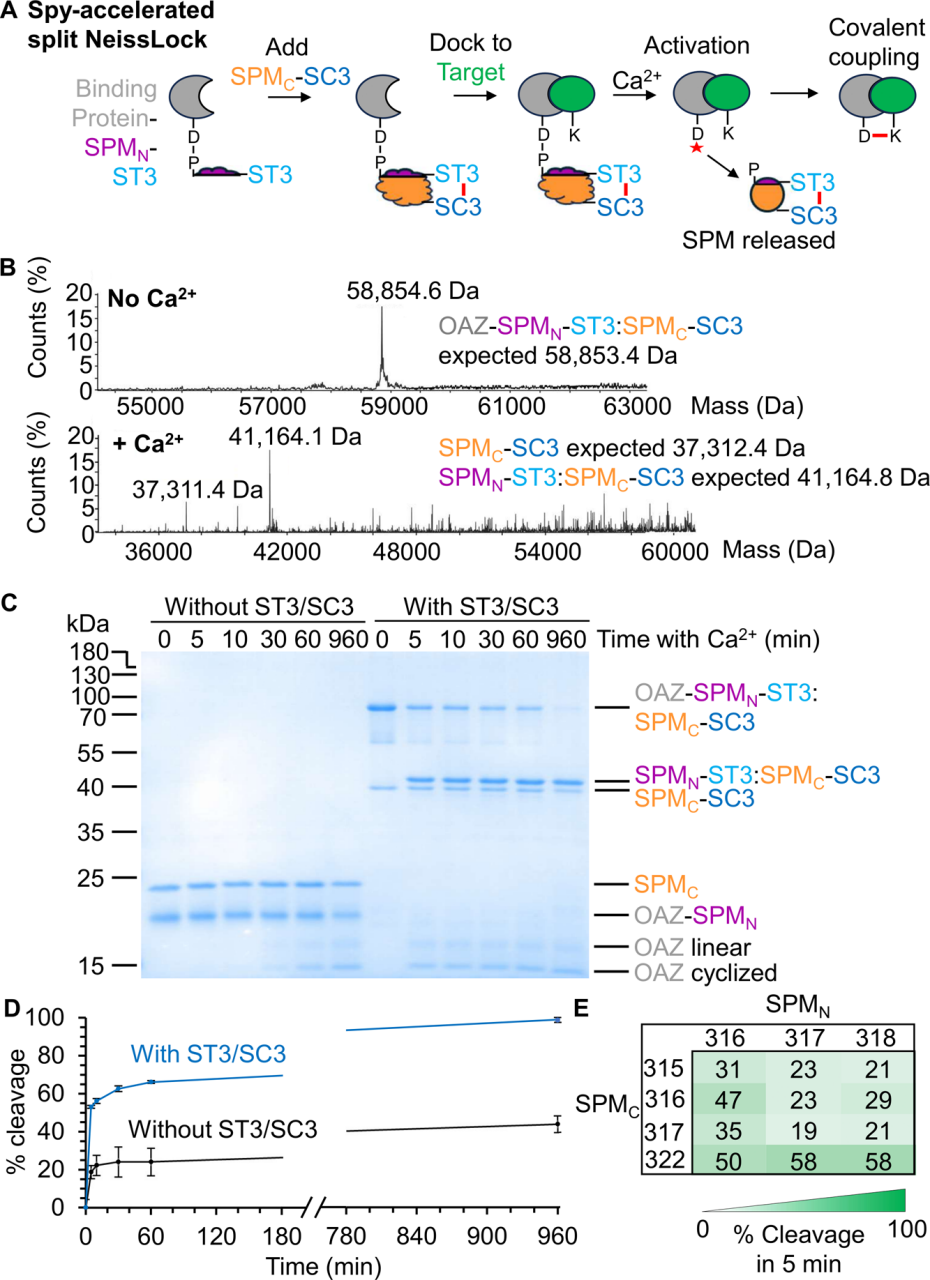
Spy-directed split NeissLock. (A) Schematic of Spy-accelerated split NeissLock. A binding protein fused to SPM’s N-terminal fragment and SpyTag003 reacts with SPM’s C-terminal fragment fused to SpyCatcher003, to promote SPM reconstitution before calcium activation. (B) Electrospray-ionization MS of reconstitution and SPM cleavage. OAZ-SPM_N_-SpyTag003 was incubated with SPM_C_-SpyCatcher003 and analyzed ± calcium. (C) Spy-acceleration of split SPM cleavage. 2 μM OAZ-SPM_N_ was incubated with 2 μM SPM_C_ ± SpyTag003/SpyCatcher003 fusion at 37 °C, before adding calcium for the indicated time and SDS-PAGE/Coomassie. (D) Quantification of Spy-accelerated cleavage, based on (C) (mean ± 1 s.d., *n* = 3). (E) Optimization of the Split Site for Spy-acceleration. Percentage cleavage upon mixing the OAZ-SPM_N_-SpyTag003 and SPM_C_-SpyCatcher003 variants was displayed as a heat map. 2 μM of each fragment was preincubated for 1 h at 37 °C, before calcium for 5 min (mean of *n* = 3).

OAZ-SPM_N_ was mixed with SPM_C_ with or without SpyTag003/SpyCatcher003. With Spy-assistance, 53 ± 4.3% OAZ was cleaved after 5 min and 92 ± 0.7% after 16 h (mean ± 1 s.d., *n* = 3). Without SpyTag003/SpyCatcher003, 19 ± 3.5% was cleaved in 5 min and only 41 ± 4.0% after 16 h (Figure 3C/D) (mean ± 1 s.d., *n* = 3). Hence, Spy ligation greatly accelerated split FrpA cleavage. We explored different SPM fragment lengths for further optimization of cleavage speed, but the same split sites (SPM_N_318 and SPM_C_322) were optimal (Figure 3E, amino acid sequences provided in Figure S6).

### Split FrpA Coupling of Tissue Plasminogen Activator or Cytokine Domains to the Mammalian Cell Surface

Covalently attaching therapeutic proteins at the surface of cells has the potential for improving therapeutic efficacy and pharmacokinetics. We selected the well-studied interaction between Transforming Growth Factor-α (TGFα) and EGFR for optimizing split NeissLock coupling to cells. We previously showed that NeissLock could drive covalent ligation of TGFα to EGFR on A431 cells, a human carcinoma cell line.^17^ As a model therapeutic to attach, we chose tissue plasminogen activator (tPA), which cleaves plasminogen to plasmin to help degrade fibrin clots as an antithrombolytic treatment for stroke^25^ and myocardial infarction.^26^ Given the importance of glycosylation of tPA for activity and stability,^27^ we expressed tPA-TGFα-SPM_N_-SpyTag003 in human cells. In this construct, tPA is the model therapeutic, TGFα is the binding protein directing the interaction with EGFR, and SPM_N_-SpyTag003 is the module for split NeissLock activation. This multimodule construct was efficiently expressed and purified by SpySwitch chromatography^28^ (Figure S7), with glycosylation confirmed by Peptide N-Glycosidase F (PNGase F) digestion (Figure S8). For pilot experiments, we first tested coupling to the soluble extracellular region of EGFR (sEGFR). Previously, we showed by MS/MS that NeissLock-activated TGFα reacted with K465 of sEGFR, the closest amine to the C-terminus of TGFα.^17^ We employed Spy-directed split FrpA reconstitution and added sEGFR, before activation with calcium. Using the recombinant soluble EGFR ectodomain, we observed the desired formation of the tPA-TGFα-SPM_N_-SpyTag003:SPM_C_-SpyCatcher003 complex, cleavage to release SPM_N_-SpyTag003:SPM_C_-SpyCatcher003, and formation of the tPA-TGFα:sEGFR product (Figure 4A).

**Figure 4:**
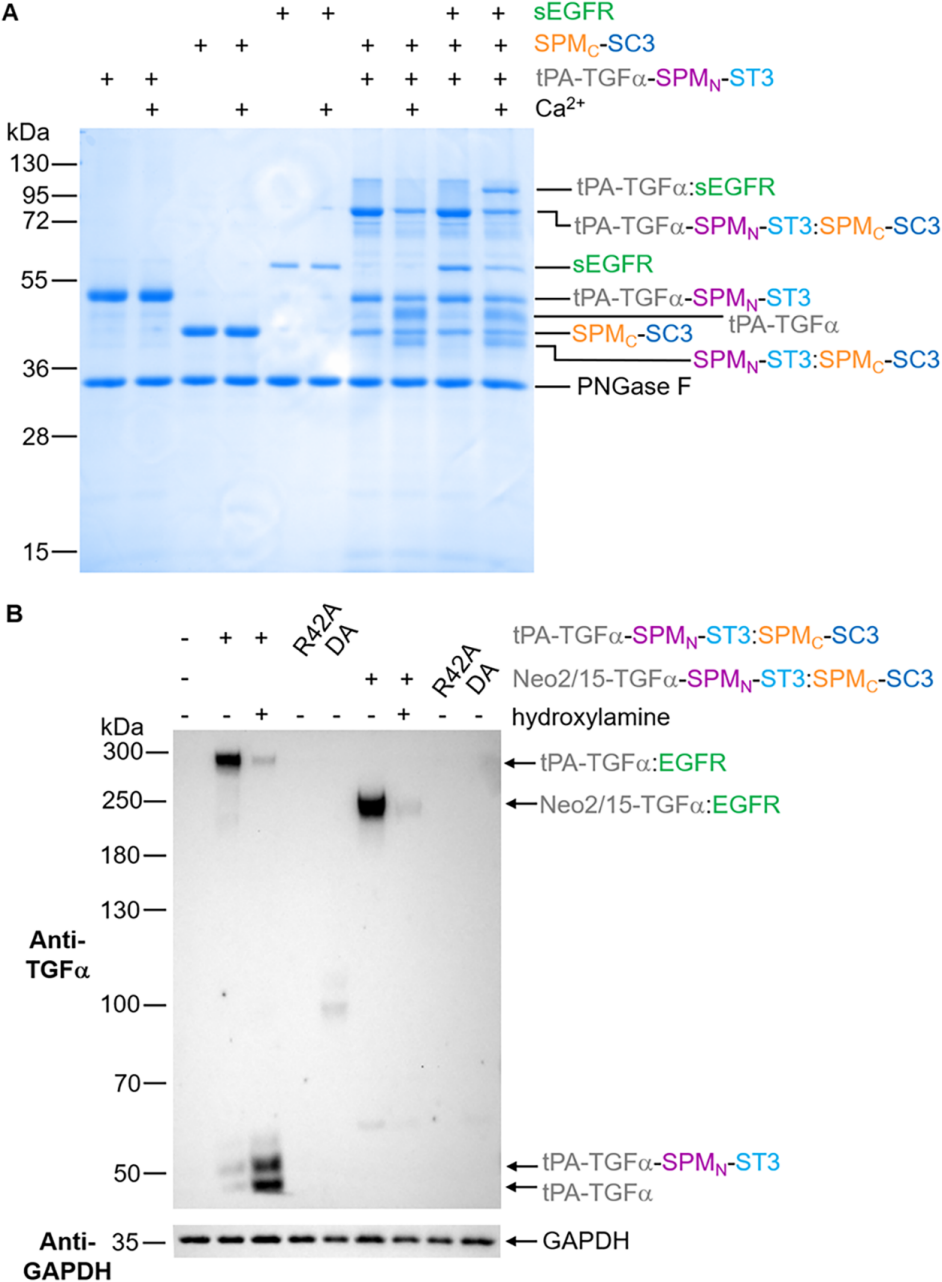
Spy-directed split NeissLock for coupling of model therapeutic domains to EGFR. (A) Covalent ligation of tPA to EGFR’s extracellular domain. 3 μM tPA-TGFα-SPM_N_-SpyTag003 was reconstituted with 3 μM SPM_C_-SpyCatcher003 for 30 min at 25 °C. 1.4 μM amount of sEGFR (the soluble extracellular region of EGFR) was added for 15 min at 37 °C, followed by Ca^2+^ for 1 h at 37 °C. Coupling was analyzed by SDS-PAGE/Coomassie, after PNGase F deglycosylation to simplify banding patterns. (B) Specific coupling of tPA or cytokine domains to EGFR in living cells. A431 cells were labeled for 10 min with 2 mM calcium and 1 μM tPA or Neo2/15 linked to TGFα for split NeissLock. Covalent products were detected by Western blot with anti-TGFα. Controls have R42A TGFα to block EGFR binding, DA-mutated SPM_N_ to block anhydride formation, or hydroxylamine to inactivate the anhydride. Blotting to GAPDH was the sample processing control.

We next tested coupling to endogenous EGFR at the surface of living human cells. To demonstrate the versatility of the split NeissLock approach, in addition to tPA, we also tested the coupling to cells of a second therapeutic, an interleukin-2 (IL-2) mimetic. IL-2 shows promise as a cancer therapeutic or antiviral, but life-threatening systemic toxicity has limited its use.^29^ We chose to use the computationally designed Neo2/15 protein that retains high affinity for IL-2 receptor βγ_c_ chains, but does not bind IL-2Rα or IL-15α to decrease toxicity.^29^ Neo2/15 has led to enhanced therapeutic activity in models of melanoma and colon cancer compared to IL-2.^29^ We genetically fused Neo2/15 to TGFα-SPM_N_-SpyTag003, before expression in human cells and purification by SpySwitch chromatography. With split SPM, no precleavage of the Neo2/15 construct was observed during Expi293F expression (Figure S9), whereas the equivalent Neo2/15 construct with full-length SPM was almost completely cleaved by Expi293F cells (Figure S10). After reconstitution of tPA or Neo2/15 linked to TGFa-SPM_N_-SpyTag003 with bacterially expressed SPM_C_-SpyCatcher003 for 1 h at 25 °C, coupling of tPA or Neo2/15 to A431 cells was activated by the addition of 2 mM calcium. Cells were incubated with the proteins and calcium for 10 min at 37 °C, before subsequent washes to remove unbound proteins. Detecting by anti-TGFα Western blot, only one product band was formed on cells, consistent with the expected molecular weight of tPA or Neo2/15 fused to TGFα:EGFR, illustrating the high specificity of split NeissLock coupling (Figure 4B).

For both tPA and Neo2/15 constructs, the coupling to EGFR was almost completely abolished where hydroxylamine quenched the anhydride (Figure 4B). Hydroxylamine is a strong nucleophile that would outcompete protein nucleophiles in reacting with the cyclic anhydride.^17^ This result supports the dependence of coupling on the anhydride formation. Similarly, mutating the reactive Asp in SPM to Ala (DA) blocked anhydride formation,^17^ and consequently, no coupling to EGFR was observed (Figure 4B). Finally, introducing the R42A mutation to TGFα, which disrupts binding to EGFR,^30^ abolished coupling to EGFR (Figure 4B). This result is consistent with the dependence on initial noncovalent EGFR binding for directing NeissLock-mediated coupling.

## Discussion

In summary, we have established unique characteristics of split NeissLock for covalent coupling to living cells, based on 3 advances. First, we identified how the SPM from FrpA provides an ultrafast module for anhydride formation. Second, we showed how an SPM could be dissected into a short peptide and protein partner, creating a new layer of inducibility and enabling eukaryotic expression of complex post-translationally modified building blocks for anhydride-mediated ligation. Third, we established the integration of split NeissLock with the rapid reactivity of SpyTag003/SpyCatcher003. Spy-directed split NeissLock is applicable for specific labeling under cell-compatible conditions within 10 min. Like SPM, SpyTag003/SpyCatcher003 is released from the final complex between the binding protein and the target protein following autoproteolysis. Split NeissLock avoids coupling methodologies involving ultraviolet light or free radical generation,^31^ likely to cause toxicity, and avoids the complexity of noncanonical amino acid mutagenesis.^32^ We demonstrated modularity by NeissLock coupling with an unmodified cellular receptor using both a therapeutic enzyme and a computationally designed cytokine. There are two regioisomers that can result from attack on a cyclic anhydride, but the regioselectivity in NeissLock is very hard to determine. Previous studies on aspartyl anhydrides showed that attack may occur at either carbonyl, with regioselectivity highly sensitive to solvent polarity and nucleophile identity.^33^

Cell therapy is delivering major impact, following clinical successes for CAR-T cells^34^ and stem cells.^35^ Although CAR-T cells have been approved for patients with B-cell malignancies or relapsed and/or refractory multiple myeloma, CAR-T cells have shown limited efficacy against most solid tumors, highlighting the need for strategies to enhance CAR-T cell efficacy so that more patients may benefit.^36^ One such strategy is arming CAR-T cells with cytokines like IL-2 or IL-15 to enhance the potency as well as persistence of CAR-T cells.^37,38^ To do so, CAR-T cells are usually genetically modified to express and secrete immunomodulatory cytokines for local delivery. However, genetic modification adds cost and prolongs the manufacturing time of CAR-T cells. Since it does not require genetic modification, we envision using split NeissLock as a fast and facile way to couple cytokines to CAR-T cells preinfusion. This could improve the CAR-T cell effector function, activate the endogenous immune system, and enhance overall immunotherapy efficacy.

Another possible application of split NeissLock is coupling therapeutic enzymes to red blood cell carriers as circulating bioreactors, capitalizing on the ∼120 day circulation time of red blood cells.^39^ For instance, tPA coupled to red blood cells has potential for treating patients with acute ischemic strokes.^40^ Alternatively, coupling enzymes to red blood cells could help patients with orphan diseases like severe combined immunodeficiency from adenosine deaminase deficiency,^41^ or Mitochondrial Neurogastrointestinal Encephalomyopathy (MNGIE) from thymidine phosphorylase deficiency.^42^ Currently, patients with metabolic deficiencies require regular intravenous enzyme replacement therapy infusions. By coupling enzymes to red blood cells, the frequency of the infusions could be reduced. Currently, few methods are available to modify the surface of red blood cells while preserving red blood cell function.^39,43^ Split NeissLock could be used to engineer red blood cells to carry therapeutic enzymes with minimal impact on the integrity of the plasma membrane. All in all, it is vital to advance the engineering of highly reactive proteins like split NeissLock to fulfill the potential of modular cell decoration.

## Supplementary Material



## Notes

Additional materials and methods; amino acid alignment of SPM homologs; and amino acid sequences of finalized split NeissLock pair compared to parental FrpA SPM (PDF)

## §

Institute of Molecular and Cell Biology, Agency for Science, Technology and Research (A*STAR), 61 Biopolis Drive, Singapore 138673, Singapore
