# Supplementary material for: Split NeissLock with Spy-Acceleration Arms Mammalian Proteins for Anhydride-Mediated Cell Ligation
Source: ACS Chem Biol. 2025 Sep 15;20(10):2475–82. doi: 10.1021/acschembio.5c00515 (PMC12538545; doi:10.1021/acschembio.5c00515)
Supplement: Supplementary file 1 [file cb5c00515_si_001.pdf]

Supporting information for:

Split NeissLock with Spy-acceleration arms mammalian proteins for anhydride-mediated cell ligation

Sheryl Y.T. Lim[1,2*], Anthony H. Keeble[3*] and Mark R. Howarth[1,3†]

[1]Department of Biochemistry, University of Oxford, South Parks Road, Oxford OX1 3QU, U.K.
[2]Current address: Institute of Molecular and Cell Biology, Agency for Science, Technology and Research (A*STAR), 61 Biopolis Drive, Singapore 138673, Singapore
[3]Department of Pharmacology, University of Cambridge, Tennis Court Road, Cambridge CB2 1PD, U.K.
* S.Y.T.L. and A.H.K. contributed equally to this work

†Corresponding author and Lead Contact:
Mark R. Howarth,
Department of Pharmacology,
University of Cambridge,
Tennis Court Road,
Cambridge,
CB2 1PD,
U.K.
E-mail: mh2186@cam.ac.uk

Materials and Methods

Bacterial strains

E. coli NEB Turbo cells (New England Biolabs) were used to amplify plasmids and were grown in LB medium with antibiotic at 37 °C. Proteins were expressed in *E. coli* BL21(DE3) RIPL (Agilent).

Cell-lines

Mammalian proteins were expressed in Expi293F cells (Thermo Fisher), maintained in Expi293 Expression media (Thermo Fisher) supplemented with 50 U/mL penicillin/streptomycin (Thermo Fisher). Cells were grown in a humidified Multitron Cell incubator (Infors HT) at 37 °C with 7% (v/v) CO_2, rotating at 110 to 125 rpm. A431 cells, a human epidermoid carcinoma cell-line from Cancer Research UK, Lincoln's Inn Fields, were grown in complete media [Dulbecco's Modified Eagle Medium–high glucose (DMEM) supplemented with 10% (v/v) fetal bovine serum (FBS), penicillin/streptomycin (Gibco, 100 U/mL), and 1× GlutaMAX (Gibco)] at 37 °C, with 5% (v/v) CO_2.

Plasmids and cloning

Plasmids were constructed using PCR-based cloning methods with Q5 High-Fidelity 2× master mix (New England Biolabs) or KOD polymerase (EMD Millipore), followed by Gibson assembly. Synthetic gene fragments were either codon-optimized for expression in *E. coli* and ordered from Integrated DNA Technologies before cloning into the pET28a backbone for bacterial expression, or codon-optimized for expression in mammalian cells before cloning into pcDNA3.1 or pENTR4 backbones for mammalian expression. The open-reading frames of all constructs were validated by Sanger sequencing (Source Bioscience).

Human ornithine decarboxylase 1 (ODC) was cloned as pET28a-His_6-ODC-Ctag (GenBank MW364944, Addgene plasmid #163614) and previously described[1]. Residues 95-219 of human OAZ (UniProt P54368) with sequence numbering based on OAZ/ODC crystal structure (PDB 4ZGY)[2] was used. Residue numbers for the FrpC self-processing module (SPM) span amino acid residues 414–657 of FrpC from *N. meningitidis* serogroup B (strain MC58) (UniProt Q9JYV5)[3]. This FrpC SPM was cloned as pET28a-AviTag-His_6-OAZ-GSY-SPM-Ctag with a mutation (C175A) in OAZ to minimize disulfide-based aggregation[1]. This construct formed the basis for the cloning of alternative SPM domains. pET28a-AviTag-His_6-OAZ-SPM(FrpA)-Ctag (GenBank PX255652, Addgene plasmid #246646) was cloned using the FrpA SPM based on position 298-543 of FrpA from *Neisseria meningitidis* serotype C (UniProt P55126). pET28a-AviTag-His_6-OAZ-SPM(*A. filiformis*)-Ctag (GenBank PX255653) was cloned using the SPM based on residues 231-476 of hemolysin-type calcium-binding protein related domain-containing protein from *Alysiella filiformis* (GenBank: SOD68839.1). pET28a-AviTag-His_6-OAZ-SPM(*K. negevensis*)-Ctag (GenBank PX255654) was cloned using the SPM based on residues 190-434 of the bifunctional hemolysin/adenylate cyclase precursor from *Kingella negevensis* (GenBank: SNB83338.1). The native -1 and -2 residues relative to the cleavable Asp-Pro bond were retained in these constructs to promote efficient activity[1]. Split FrpA SPM N-terminal variants were cloned from pET28a-AviTag-His_6-OAZ-SPM(FrpA)-Ctag with differing lengths to the SPM (C-terminal deletions) and with the C-tag deleted. Constructs pET28a-AviTag-His_6-OAZ-SPM(FrpA 298-315), pET28a-AviTag-His_6-OAZ-SPM(FrpA 298-318), and pET28a-AviTag-His_6-OAZ-SPM(FrpA 298-321) were cloned in a similar manner, with the numbers referring to the residues of the N-terminal part of the SPM retained in the construct. Constructs pET28a-AviTag-His_6-OAZ-SPM(FrpA 298-316)-GSG-SpyTag003, pET28a-AviTag-His_6-OAZ-SPM(FrpA 298-317)-GSG-SpyTag003 and pET28a-AviTag-His_6-OAZ-SPM(FrpA 298-318)-GSG-SpyTag003 (GenBank PX255655,

Addgene plasmid # 246647) contain an extra GSG spacer followed by a SpyTag003 at the C-terminus. C-terminal variants were cloned from pET28a-AviTag-His_6-OAZ-SPM(FrpA)-Ctag with differing lengths to the SPM (N-terminal deletions) and retained C-tag. Constructs pET28a-SPM(FrpA 315-543)-Ctag, pET28a-SPM(FrpA 316-543)-Ctag, pET28a-SPM(FrpA 317-543)-Ctag, pET28a-SPM(FrpA 319-543)-Ctag, and pET28a-SPM(FrpA 322-543)-Ctag were cloned with the numbers referring to the residues of the C-terminal part of the SPM retained in the construct. Variants with SpyCatcher003 domains following the C-terminal variants were cloned from these plasmids with the arrangement SPM(FrpA C-term portion)-GSGAM linker-SpyCatcher003-GSSGSGSG-Ctag. Constructs pET28a-SPM(FrpA 315-543)-SpyCatcher003-Ctag, pET28a-SPM(FrpA 316-543)-SpyCatcher003-Ctag, pET28a-SPM(FrpA 317-543)-SpyCatcher003-Ctag, and pET28a-SPM(FrpA 322-543)-SpyCatcher003-Ctag were cloned in this manner. pET28a-His_6-SUMO-SPM_C-SpyCatcher003-Ctag (GenBank PX255656, Addgene plasmid # 246648) was cloned from pET28a-SPM(FrpA 322-543)-SpyCatcher003-Ctag. pENTR4-sEGFR-His_6, encoding a soluble fragment of the extracellular domain of human EGFR (UniProt P00533, residues 25–525), has been described[1]. pOPINE His_6- SUMO protease Ulp1 has been described[4]. pcDNA3.1 AP-His_6-sfGFP-SPM was cloned with the domain organization: tPA signal peptide, AviTag biotinylation peptide-His_6-sfGFP-FrpA full-length SPM. pcDNA3.1 tPA-TGFα-SPM_N-SpyTag003 (GenBank PX255657, Addgene plasmid # 246649) was cloned with the organization: tPA signal peptide, Kringle domain of human tPA, protease domain of human tPA containing the mutations found in Tenectaplase (K296A, H297A, R298A and R299A) that result in enhanced resistance to the plasma inhibitor PAI-1[5], GSS linker, TGFα (residues 40–89 of human protransforming growth factor alpha), SPM(FrpA 298-318), GSG linker, SpyTag003. pcDNA3.1 tPA-TGFα (R42A)-SPM_N-SpyTag003 was cloned from pcDNA3.1 tPA-TGFα-SPM_N-SpyTag003 with mutation of Arg42 of the TGFα sequence to alanine[6]. pcDNA3.1 tPA-TGFα-SPM_N DA-SpyTag003 was cloned from pcDNA3.1 tPA-TGFα-SPM_N-SpyTag003 with mutation at the aspartic acid residue that forms the anhydride. pcDNA3.1 Neo2/15-TGFα-SPM_N-SpyTag003 (GenBank PX255658, Addgene plasmid # 246650) was cloned with the following organization: tPA signal peptide, Neo2/15[7], GSS linker, TGFα (residues 40–89 of human protransforming growth factor alpha), GSY, SPM(FrpA 298-318), GSG linker, SpyTag003. pcDNA3.1 Neo2/15-TGFα (R42A)-SPM_N-SpyTag003 was cloned from pcDNA3.1 Neo2/15-TGFα-SPM_N-SpyTag003 with mutation of Arg42 of the TGFα sequence to alanine[6]. pcDNA3.1 Neo2/15-TGFα-SPM_N DA-SpyTag003 was cloned from pcDNA3.1 Neo2/15-TGFα-SPM_N-SpyTag003 with mutation at the aspartic acid residue that forms the anhydride. pcDNA3.1 Neo2/15-TGFα-SPM-His_6 was cloned from pcDNA3.1 Neo2/15-TGFα-SPM_N-SpyTag003 by cloning FrpA SPM-His_6 in place of SPM_N-SpyTag003.

Bacterial protein expression and purification

Bacterial expression was in *E. coli* BL21(DE3) RIPL cells. After transformation of the plasmids into the growth strain, the cells were recovered on LB agar plates + 50 µg/mL kanamycin overnight at 37 °C. Single colonies were picked into starter cultures of 10 mL LB containing 25 µg/mL kanamycin and grown for 16 h at 37 °C with shaking at 200 rpm. Expression cultures were inoculated with 1/100 dilution of the saturated starter in 1 L LB + 0.8% (w/v) glucose [for expressions in BL21(DE3) RIPL] supplemented with 25 µg/mL kanamycin in ultra-yield baffled flasks (Thomson Instrument Company). Cultures were grown at 37 °C with shaking at 200 rpm until A_{600} 0.5 and protein expression was induced with 0.42 mM isopropyl β-D-1-thiogalactopyranoside (IPTG) with shaking at 200 rpm at 25 °C for 18 h. Cells were harvested by centrifugation and processed immediately or stored at -80 °C.

For proteins without His_6-tags but containing C-tags, cells were resuspended in ice-cold bacterial lysis buffer [30 mM Tris-HCl pH 7.4, 200 mM NaCl, 5% (v/v) glycerol, cOmplete

mini EDTA-free protease inhibitor cocktail, 1 mM phenylmethylsulfonyl fluoride (PMSF), 1 mg/mL lysozyme (Merck) and 2 U/mL benzonase (Merck)]. Cells were lysed by sonication on ice, with the cells sonicated 3–4 times for 1 min at 50% duty cycle with 1 min rest periods. Cell lysate was clarified for 30 min by centrifugation in a JA25−50 rotor (Beckman) at 30,000–35,000 g at 4 °C. The clarified lysate was incubated with CaptureSelect C-tagXL Affinity Matrix (Thermo Fisher) resin pre-equilibrated with C-tag binding/wash buffer (30 mM Tris-HCl pH 7.4, 200 mM NaCl) in a Poly-Prep gravity column and mixed by rotation for 30-60 min at 4 °C. The column was drained, the resin washed with binding/wash buffer, and proteins eluted in 50 mM HEPES plus 2 M $MgCl_2$, pH 7.4, with all washes and elution steps at 4 °C. Protein concentrations were determined from A_{280} using the extinction coefficient from ExPASy ProtParam. Typical yields were 8-15 mg/L culture.

For proteins purified by His_6-tag, cells were resuspended in ice-cold bacterial lysis buffer supplemented with 10 mM imidazole, pH 7.4. Cells were lysed by sonication on ice, with the cells sonicated 3–4 times for 1 min at 50% duty cycle with 1 min rest periods. Cell lysate was clarified for 45 min by centrifugation in a JA25−50 rotor (Beckman) at 30,000–35,000 g at 4 °C. The clarified lysate was incubated with Ni-NTA resin (Qiagen) pre-equilibrated with Ni-NTA binding/wash buffer (30 mM Tris-HCl, 200 mM NaCl, 10 mM imidazole pH 7.4) in a Poly-Prep gravity column and mixed by rotation for 30-60 min at 4 °C. The column was drained and the resin was washed with binding/wash buffer, before eluting in 30 mM Tris-HCl pH 7.4, 200 mM NaCl, plus 200 mM imidazole at 4 °C. When purifying ODC or OAZ variants, 5 mM 2-mercaptoethanol was supplemented in wash and elution buffers. Typical yields were 8-15 mg/L culture.

After either His_6-tag or C-tag purification, eluted proteins were concentrated using a Vivaspin centrifugal concentrator with 10 or 30 kDa cut-off (GE Healthcare) and centrifuged at 4,000 g at 4 °C. A HiLoad 16/600 Superdex 200 pg column (GE Healthcare) was connected to the ÄKTA pure 25 fast protein liquid chromatography (FPLC) system (GE Healthcare) for size-exclusion chromatography (SEC). The column was pre-equilibrated with running buffer [50 mM HEPES pH 7.4, 150 mM NaCl, 2 mM tris(2-carboxyethyl)phosphine (TCEP)] for gel filtration of OAZ and ODC. When purifying ODC variants, the running buffer was supplemented with an additional 0.02 mM pyridoxal phosphate (Sigma-Aldrich) as co-factor. Proteins samples were loaded at 1 mL/min and isocratic elution was carried out with 1 mL fractions collected. Based on the A_{280} peak, fractions of interest were collected and the purity of the fractions was verified by SDS-PAGE/Coomassie. Fractions containing the protein of interest were concentrated to ~500 µL using a Vivaspin centrifugal concentrator with 10 or 30 kDa cut-off (GE Healthcare). Long-term storage of the proteins was at -80 °C.

For SUMO domain removal, His_6-SUMO-SPM_C-SpyCatcher003-Ctag was dialyzed into TBS pH 7.5 (50 mM Tris-HCl pH 7.5, 150 mM NaCl) and concentrated using a Vivaspin centrifugal concentrator with a 10 kDa cut-off (GE Healthcare). SUMO protease Ulp1[4] was added at 1:50 molar ratio, followed by 45 min incubation at 25 °C. Remaining His_6-tagged proteins (SUMO and Ulp1) in a volume of 800 µL were incubated with 200 µL Ni-NTA (Qiagen) with rotation for 1 h on a tube rotor at 25 °C, followed by application to a Polyprep column and collection of the column flow-through containing the SPM_C-SpyCatcher003-Ctag.

Mammalian protein expression and purification

Neo2/15-TGFα-SPM_N-SpyTag003 variants, tPA-TGFα-SPM_N-SpyTag003 variants, and sEGFR-His_6 were expressed by transient transfection in Expi293F cells (Thermo Fisher). Cells were grown in a humidified Multitron Cell incubator (Infors HT) at 37 °C with 8% (v/v) CO_2, rotating at 95 rpm to 3.0×10^6 cells/mL in Expi293 expression media supplemented with 50 U/mL penicillin/streptomycin (Thermo Fisher). Cells were transferred into Expi293 expression media with no antibiotics present and transfected with plasmid using the ExpiFectamine 293

Transfection Kit (Thermo Fisher). Plasmid DNA (1 μg per mL of culture) was incubated with ExpiFectamine 293 reagent for 20 min and then added dropwise to the Expi293F cells. Cells were grown for 20 h before addition of ExpiFectamine 293 Transfection Enhancers 1 and 2. When expressing sEGFR, kifunensine (Sigma-Aldrich) was added to the culture concurrent to transfection to a final 5 μM to promote homogeneous glycosylation. Proteins were harvested 4-8 days post-transfection.

Proteins containing a His_6-tag were purified by Ni-NTA (Qiagen). The cell supernatant was harvested and then we added 1/10th volume of 10× Ni-NTA binding buffer, mixed protease inhibitors (cOmplete mini EDTA-free protease inhibitor cocktail, Roche) and imidazole to a final 10 mM. Resin was pre-equilibrated with Ni-NTA binding/wash buffer (30 mM Tris-HCl, 200 mM NaCl, 10 mM imidazole, pH 7.4) and incubated with the cell supernatant in a Poly-Prep gravity column and mixed by rotation for 30-60 min at 4 °C. The column was drained, the resin was washed with Ni-NTA binding/wash buffer, and proteins were eluted in 30 mM Tris-HCl, 200 mM NaCl, 200 mM imidazole, pH 7.4 at 4 °C.

Proteins containing SpyTag003 but no His_6-tag were purified by SpySwitch[8]. The cell supernatant was harvested and 1/10th volume of 10× SpySwitch buffer (500 mM Tris-HCl pH 7.5 + 3 M NaCl) was added, supplemented with mixed protease inhibitors (cOmplete mini EDTA-free protease inhibitor cocktail, Roche). Resin was pre-equilibrated with SpySwitch binding buffer (50 mM Tris-HCl pH 7.5, 300 mM NaCl) and incubated with the cell supernatant in a Poly-Prep gravity column, with mixing by rotation for 30-60 min at 4 °C. The column was drained and the resin was washed with SpySwitch buffer at 4 °C. Proteins were eluted with 1.5 column volumes of SpySwitch elution buffer (50 mM acetic acid/sodium acetate pH 5.0, 150 mM NaCl) at 4 °C and the elution was collected into a microcentrifuge tube containing 0.3 column volumes of 1 M Tris-HCl pH 8.0, with the microcentrifuge tube mixed by inversion to minimize time spent at an acidic pH. Typical yields of proteins were 4-8 mg/L culture.

Polyacrylamide gel electrophoresis

SDS-PAGE was performed using 7-14% polyacrylamide gels in an XCell SureLock system (Thermo Scientific). Electrophoresis was carried out at 180-200 V in 25 mM Tris-HCl pH 8.5, 192 mM glycine, 0.1% (w/v) SDS. For 7% Tris-Acetate gels, electrophoresis was carried at 180 V in 50 mM Tricine, 50 mM Tris base, pH 8.24, 0.1% (w/v) SDS. Gels were stained with Brilliant Blue G-250 and destained with Milli-Q H_2O, prior to imaging on a ChemiDoc XRS+ imager. ImageLab 6.1.0 software (Bio-Rad) was used for densitometric quantification. For imaging on an iBright FL1500 imaging system (Thermo Fisher), analysis was performed using iBright Analysis Software Versions 5.0.1 and 5.2.0 (Thermo Fisher). On gels, a colon between proteins indicates conjugates that are hypothesized to be covalently coupled.

SPM cleavage and protein conjugation assays

Reactions were carried out at 37 °C (except when otherwise stated) in HEPES-buffered saline (HBS: 50 mM HEPES pH 7.4, 150 mM NaCl), supplemented with 2 mM TCEP when ODC proteins were included. Cleavage of SPM was initiated by addition of pre-warmed reaction buffer containing calcium chloride to the required concentration. Reactions were incubated for the desired time and quenched by addition of EDTA, to chelate Ca^{2+}, in 5× SDS-loading buffer [0.19 M Tris-HCl pH 6.8, 20% (v/v) glycerol, 100 μM bromophenol blue, 0.19 M SDS] with EDTA at 15 mM and heating at 95 °C for 5 min in a C1000 Touch Thermal Cycler (Bio-Rad). The degree of SPM cleavage and coupling was assessed by SDS-PAGE/Coomassie densitometry. Assays were run in triplicate and the percentage cleavage/coupling expressed as the mean ± 1 S.D. from n=3.

Cleavage and coupling reactions of ODC with SPM homologs were carried out with 5 μM ODC pre-mixed with 5 μM OAZ-SPM variants in HBS + 2 mM TCEP at 37 °C. Reaction

was activated by addition of a final concentration of 10 mM $CaCl_2$. Reaction aliquots were sampled across a range of time-points.

Detailed comparison of the cleavage and coupling reactions between ODC and FrpC or FrpA SPMs was repeated with modified conditions. 10 μM ODC was pre-mixed with either OAZ-SPM(FrpC) or OAZ-SPM(FrpA) in HBS + 2 mM TCEP at 25 °C and reaction was activated by addition of a final concentration of 1 mM $CaCl_2$. Reaction aliquots were sampled across a range of time-points.

Initial comparison of the split FrpA calcium-induced cleavage and coupling to ODC was carried out with 10 μM of OAZ-SPM-N terminal variant incubated with 10 μM of SPM-C terminal variant with or without 10 μM ODC and 10 mM Ca^{2+} in HBS + 2 mM TCEP, at 37 °C for 16 h. Time-courses for the conjugation to ODC with different split variants were repeated with activation by 10 mM Ca^{2+} in HBS + 2 mM TCEP at 37 °C, but with 5 μM OAZ-SPM_N terminal variant incubated with 5 μM SPM_C terminal variant and 5 μM ODC before Ca^{2+} activation. Reaction aliquots were sampled across a range of time-points.

For comparison of the impact of proximity labeling with SpyTag003/SpyCatcher003 on Ca^{2+}-induced cleavage of the optimized split FrpA SPM, the two half-proteins were mixed and incubated in HBS at 37 °C for 1 h. In parallel experiments 2 μM OAZ-SPM_N318 was incubated with 2 μM SPM_C322, whilst 2 μM OAZ-SPM_N318-SpyTag003 was incubated with 2 μM SPM_C322-SpyCatcher003. 10 mM Ca^{2+} was then added to initiate cleavage and reaction aliquots were sampled across a range of time-points.

For coupling of SPM-reconstituted Neo2/15-TGFα-SPM_N-SpyTag003 and tPA-TGFα-SPM_N-SpyTag003 with sEGFR, reactions were carried out in HBS. In parallel reactions, 3 μM SPM_C-SpyCatcher003-Ctag was incubated with 3 μM Neo2/15-TGFα-SPM_N-SpyTag003 and tPA-TGFα-SPM_N-SpyTag003 for 30 min at 37 °C. 1.54 μM sEGFR was then added and equilibrated for 30 min at 37 °C, before activation with a final 2 mM Ca^{2+} for 1 h. Control reactions without Ca^{2+} or sEGFR were also carried out. Post-reaction samples were digested with Peptide:N-glycosidase F (PNGase F) under denaturing conditions. Glycoprotein Denaturing Buffer (10× stock, NEB) was added to a final concentration of 1×, and samples were heated for 10 min at 100 °C. After cooling to 25 °C, Glycoprotein Buffer 2 (NEB) was added to 1×, NP-10 (NEB) was added to 10% (v/v), and PNGase F (NEB) was added to 50,000 U/mL. Samples were then digested for 1 h at 37 °C before SDS-PAGE.

Calculation of SPM cleavage and coupling

The percentage SPM cleaved was calculated using Equation 1.

$$\%\,Cleavage = \left(1 - \left(\frac{Intensity\ of\ SPM\ band\ at\ time\ t}{Intensity\ of\ SPM\ band\ at\ time\ 0}\right)\right) \times 100 \qquad \text{Equation 1}$$

The percentage of ODC coupled was calculated using Equation 2.

$$\%\,ODC\ coupled = \left(1 - \left(\frac{Intensity\ of\ ODC\ band\ at\ time\ t}{Intensity\ of\ ODC\ band\ at\ time\ 0}\right)\right) \times 100 \qquad \text{Equation 2}$$

The relative conjugation between ODC and OAZ was calculated in Equation 3 as the band intensity of the OAZ:ODC conjugate divided by the band intensity of OAZ:ODC at 22 h for the most efficient reaction for the different versions of the split FrpA reacted together.

$$Relative\ \%\ conjugated = \left(\frac{OAZ{:}ODC\ at\ time\ t}{Max\ OAZ{:}ODC\ at\ 22h}\right) \times 100$$

Equation 3

Coupling of SPM proteins to EGFR on live cells

A431 cells were seeded in a 6-well plate at 5 × 10^5 cells/well and incubated at 37 °C with 5% (v/v) CO_2 in complete growth media for 24 h. Cells were then washed twice with 4 mL washes

of FBS-free complete media. A431 cells were grown serum-starved for a further 24 h at 37 °C with 5% (v/v) CO_2. Cells were then washed twice with 2 mL HBS-Mg (50 mM HEPES pH 7.4, 150 mM NaCl, 5 mM $MgCl_2$, sterile-filtered). In parallel reactions, 150 µL 2 µM Neo2/15-TGFα-SPM_N-SpyTag003 and tPA-TGFα-SPM_N-SpyTag003 were each reconstituted with 2 µM SPM_C-SpyCatcher003-Ctag for 1 h at 25 °C in HBS-Mg. These were then added to a well, with or without hydroxylamine (Sigma-Aldrich) dissolved to a final 5 mM in 2% (v/v) dimethylsulfoxide (DMSO) in HBS-Mg. Coupling was activated by the rapid subsequent addition of 150 µL 4 mM $CaCl_2$ in HBS-Mg, with or without hydroxylamine dissolved to final 5 mM. The cells were incubated with the SPM-containing proteins and 2 mM $CaCl_2$ for 10 min at 37 °C with 5% (v/v) CO_2, before removal of the protein solution. The cells were then incubated in FBS-free complete media containing 2% (v/v) DMSO, with or without 5 mM hydroxylamine for 1 h. After removal of the media, A431 cells were lysed by addition of 150 µL ice-cold modified RIPA buffer [20 mM Tris-HCl pH 7.4, 150 mM NaCl, 5 mM NaF, 1% (v/v) Triton-X-100, 0.1% (w/v) SDS, 0.5% (w/v) sodium deoxycholate], supplemented with cOmplete protease inhibitor mix, 1 mM PMSF and 1 mM sodium orthovanadate and scraping of the cells from the plate, followed by incubation for 20 min at 4 °C. The cell lysate was pipetted from the plates and the cleared lysate was produced by centrifugation at 12,000 g for 20 min at 4 °C.

Western blotting
Aliquots of cleared lysates were mixed with 6× SDS loading buffer containing 10 mM dithiothreitol and heated at 95 °C for 5 min in a C1000 Touch Thermal Cycler (Bio-Rad), before running on 7% Tris-Acetate SDS-PAGE for detection of TGFα-containing bands. After electrophoresis, gels were incubated in 20% (v/v) ethanol for 20 min. Proteins were transferred at 15 V for 13 min when using the iBlot 2 Dry Blotting System (Thermo Fisher). The membrane was incubated in blocking solution of 5 % (w/v) milk (Sigma-Aldrich) in TBS-T [50 mM Tris-HCl pH 7.4, 150 mM NaCl, 0.1% (v/v) Tween 20] for 1 h at 25 °C. When blotting for TGFα, the anti-TGFα antibody (MF9, Novus Biologicals) was added at 1:1,000 in the blocking solution and the membrane was incubated for 20 h at 4 °C. The membranes were then washed 4 × 5 min in TBS-T, before addition of the secondary antibody. Goat anti-mouse horseradish peroxidase (HRP) (A4416, Merck) was diluted to 1:1,000 in the blocking solution and the membranes were incubated with the antibody for 1 h at 25 °C, before another 4 × 5 min washes in TBS-T. Proteins were detected using SuperSignal West Pico PLUS Chemiluminescent Substrate (Thermo Fisher), measuring chemiluminescence on an iBright FL1500 imaging system (Thermo Fisher) with analysis using iBright Analysis Software Versions 5.0.1 and 5.2.0 (Thermo Fisher).

When detecting glyceraldehyde-3-phosphate dehydrogenase (GAPDH) as a sample processing control, aliquots of cleared lysates were mixed with 6× SDS loading buffer containing 10 mM dithiothreitol and heated at 95 °C for 5 min in a C1000 Touch Thermal Cycler (Bio-Rad) before running on a 14% Tris-glycine SDS-PAGE. After electrophoresis, transfer of bands to the membrane and blocking in TBS-T + 5 % (w/v) milk was performed as described above. GAPDH was detected using 1:1,000 mouse anti-GAPDH GA1R (Thermo Fisher) (20 h at 4 °C) and 1:2,000 Goat-anti-mouse-HRP (A4416, Merck) (1 h at 25 °C) both in 5% (w/v) skimmed milk in TBS-T. Proteins were detected as described above.

Mass Spectrometry
Purified protein constructs were analyzed with a jet-stream electrospray ionization intact protein mass spectrometry experiment, using a RapidFire 365 jet-stream electrospray ion source (Agilent) coupled to a 6550 Accurate-Mass Quadrupole Time-of-Flight (Q-TOF) (Agilent) mass spectrometer in positive ion mode. 10 µM protein samples were injected and

acidified to 0.9 % (v/v) formic acid. Samples were aspirated for 0.3 s before they were adsorbed onto a C4 solid-phase cartridge for extraction. Washes using 0.1% (v/v) formic acid were carried out for 5.5 s before elution onto the detector for 5.5 s. Data were analyzed using Mass Hunter Qualitative Analysis software B.07.00 (Agilent). Protein ionization was deconvoluted using the maximum entropy algorithm. Expected molecular weights for full-length proteins were calculated using ExPASy ProtParam, with the N-terminal fMet (bacterial expression) removed. For constructs where a SpyTag003 and SpyCatcher003 reacted with one another, isopeptide bond formation occurs releasing a water molecule, which was taken into account when calculating the expected molecular weight.

Graphics and structure analysis

Structures were visualized in PyMOL version 2.0.6 (DeLano Scientific), with FrpA split sites guided by the nuclear magnetic resonance structure of the Ca^{2+}-bound FrpC SPM (PDB 6SJW)[9]. The sequence alignment of the SPM variants was created using Clustal Omega. The model structure of the Ca^{2+}-bound FrpA SPM, comprising residues 298-543 of FrpA from *N. meningitidis* serotype C (UniProt P55126), was generated using AlphaFold 3[10] with the expected 4 Ca^{2+} ions selected to be included in the model. The model of the SPM_N-ST3:SPM_C-SC3 complex was generated using AlphaFold 3[10] using the finalized sequences for the constructs in Figure S6. The expected 4 Ca^{2+} ions were also included in the model.

Statistics and reproducibility

No statistical method was used to pre-determine sample sizes. No data were excluded from our analyses. Experiments were not randomized. The investigators were not blinded to allocation during the experiments and assessment of outcome.

Data availability

Sequences of key constructs are found in GenBank, as described in the section "Plasmids and cloning". Plasmids from this study have been deposited in the Addgene repository (https://www.addgene.org/Mark_Howarth/), as described in the section "Plasmids and cloning". Further information and request for resources and reagents should be directed to and will be fulfilled by the lead contact, M.R.H.

REFERENCES

(1) Scheu, A. H. A.; Lim, S. Y. T.; Metzner, F. J.; Mohammed, S.; Howarth, M. NeissLock Provides an Inducible Protein Anhydride for Covalent Targeting of Endogenous Proteins. *Nat. Commun.* **2021**, *12*, 717. https://doi.org/10.1038/s41467-021-20963-5.

(2) Wu, H.-Y.; Chen, S.-F.; Hsieh, J.-Y.; Chou, F.; Wang, Y.-H.; Lin, W.-T.; Lee, P.-Y.; Yu, Y.-J.; Lin, L.-Y.; Lin, T.-S.; Lin, C.-L.; Liu, G.-Y.; Tzeng, S.-R.; Hung, H.-C.; Chan, N.-L. Structural Basis of Antizyme-Mediated Regulation of Polyamine Homeostasis. *Proc. Natl. Acad. Sci. U.S.A.* **2015**, *112* (36), 11229–11234. https://doi.org/10.1073/pnas.1508187112.

(3) Osicka, R.; Prochazkova, K.; Sulc, M.; Linhartova, I.; Havlicek, V.; Sebo, P. A Novel "Clip-and-Link" Activity of Repeat in Toxin (RTX) Proteins from Gram-Negative Pathogens. Covalent Protein Cross-Linking by an Asp-Lys Isopeptide Bond upon Calcium-Dependent Processing at an Asp-Pro Bond. *J. Biol. Chem.* **2004**, *279*, 24944–24956. https://doi.org/10.1074/jbc.M314013200.

(4) Assenberg, R.; Delmas, O.; Graham, S. C.; Verma, A.; Berrow, N.; Stuart, D. I.; Owens, R. J.; Bourhy, H.; Grimes, J. M. Expression, Purification and Crystallization of a

Lyssavirus Matrix (M) Protein. *Acta Crystallogr. Sect. F Struct. Biol. Cryst. Commun.* **2008**, *64*, 258–262. https://doi.org/10.1107/S1744309108004557.
(5) Zhu, A.; Rajendram, P.; Tseng, E.; Coutts, S. B.; Yu, A. Y. X. Alteplase or Tenecteplase for Thrombolysis in Ischemic Stroke: An Illustrated Review. *Res. Pract. Thromb. Haemost.* **2022**, *6* (6), e12795. https://doi.org/10.1002/rth2.12795.
(6) Lazar, E.; Vicenzi, E.; Van Obberghen-Schilling, E.; Wolff, B.; Dalton, S.; Watanabe, S.; Sporn, M. B. Transforming Growth Factor Alpha: An Aromatic Side Chain at Position 38 Is Essential for Biological Activity. *Mol. Cell. Biol.* **1989**, *9* (2), 860–864. https://doi.org/10.1128/mcb.9.2.860-864.1989.
(7) Silva, D.-A.; Yu, S.; Ulge, U. Y.; Spangler, J. B.; Jude, K. M.; Labão-Almeida, C.; Ali, L. R.; Quijano-Rubio, A.; Ruterbusch, M.; Leung, I.; Biary, T.; Crowley, S. J.; Marcos, E.; Walkey, C. D.; Weitzner, B. D.; Pardo-Avila, F.; Castellanos, J.; Carter, L.; Stewart, L.; Riddell, S. R.; Pepper, M.; Bernardes, G. J. L.; Dougan, M.; Garcia, K. C.; Baker, D. De Novo Design of Potent and Selective Mimics of IL-2 and IL-15. *Nature* **2019**, *565* (7738), 186–191. https://doi.org/10.1038/s41586-018-0830-7.
(8) Vester, S. K.; Rahikainen, R.; Khairil Anuar, I. N. A.; Hills, R. A.; Tan, T. K.; Howarth, M. SpySwitch Enables pH- or Heat-Responsive Capture and Release for Plug-and-Display Nanoassembly. *Nat. Commun.* **2022**, *13* (1), 3714. https://doi.org/10.1038/s41467-022-31193-8.
(9) Kuban, V.; Macek, P.; Hritz, J.; Nechvatalova, K.; Nedbalcova, K.; Faldyna, M.; Sebo, P.; Zidek, L.; Bumba, L. Structural Basis of Ca2+-Dependent Self-Processing Activity of Repeat-in-Toxin Proteins. *mBio* **2020**, *11* (2), e00226-20. https://doi.org/10.1128/mBio.00226-20.
(10) Abramson, J.; Adler, J.; Dunger, J.; Evans, R.; Green, T.; Pritzel, A.; Ronneberger, O.; Willmore, L.; Ballard, A. J.; Bambrick, J.; Bodenstein, S. W.; Evans, D. A.; Hung, C.-C.; O'Neill, M.; Reiman, D.; Tunyasuvunakool, K.; Wu, Z.; Žemgulytė, A.; Arvaniti, E.; Beattie, C.; Bertolli, O.; Bridgland, A.; Cherepanov, A.; Congreve, M.; Cowen-Rivers, A. I.; Cowie, A.; Figurnov, M.; Fuchs, F. B.; Gladman, H.; Jain, R.; Khan, Y. A.; Low, C. M. R.; Perlin, K.; Potapenko, A.; Savy, P.; Singh, S.; Stecula, A.; Thillaisundaram, A.; Tong, C.; Yakneen, S.; Zhong, E. D.; Zielinski, M.; Žídek, A.; Bapst, V.; Kohli, P.; Jaderberg, M.; Hassabis, D.; Jumper, J. M. Accurate Structure Prediction of Biomolecular Interactions with AlphaFold 3. *Nature* **2024**, *630* (8016), 493–500. https://doi.org/10.1038/s41586-024-07487-w.

```
K. negevensis         DPLILDLDGKGIQTLAPSSI-SARFDHNADGIATATGWAAAGNGILALDLDNNGKIDSGK    58
A. filiformis         DPLALDLDGNGIQTTATAGFSGSLFDHNKDGIRTATGWVASGDGLLVRDLNGNGIIDNGG    59
FrpC (Neisseria)      DPLALDLDGDGIETVATKGFAGSLFDHTNNGIRTATGWVSADDGLLVRDLNGNGIIDNGA    59
FrpA (Neisseria)      DPLALDLDGDGIETVAAKGFAGALFDHRNQGIRTATGWVSADDGLLVRDLNGNGIIDNGA    59
                      *** *****.**:* *  .: .: ***  :** *****.::.:*:*. **:.** **.*

K. negevensis         EIFGNHSVLSNGTAAAHGYAALAELDSNADGIISALDDTFSSLKVWQDINQDGISQSNEL    118
A. filiformis         ELFGENTLLADGTLAQHGYAALAELDSNADGVVDANDAAFATLRVWQDKNQDGISQADEL    119
FrpC (Neisseria)      ELFGDNTKLADGSFAKHGYAALAELDSNGDNIINAADAAFQTLRVWQDLNQDGISQANEL    119
FrpA (Neisseria)      ELFGDNTKLADGSFAKHGYAALAELDSNGDNIINAADAAFQTLRVWQDLNQDGISQANEL    119
                      *:**::: *::*: * ************.*.::.* * :* :*:**** *******::**

K. negevensis         FTLQALGIQSLNLEHQENSKDLGNGNRLTHIGSYTKTDGTTGEMGDVEFASNSLYSRYTD    178
A. filiformis         HTLADLGIQSLNTAYEDVNQDLGNGNSIAQLGSYTKTDGSTAEMADLLFHNDHLYSRFAE    179
FrpC (Neisseria)      RTLEELGIQSLDLAYKDVNKNLGNGNTLAQQGSYTKTDGTTAKMGDLLLAADNLHSRFKD    179
FrpA (Neisseria)      RTLEELGIQSLDLAYKDVNKNLGNGNTLAQQGSYTKTDGTTAKMGDLLLAADNLHSRFKD    179
                       **  ******:  ::: .::***** ::: ********:*.:*.*: :  : *:**: :

K. negevensis         TIELTPEQLQAPNLHGTGRLRDLREAAALSTGLAEILKQYSAAQTKEEQTALLSELVAKW    238
A. filiformis         RIELTAEQSRAANLSGIGRVRDLREAAALFGDLSATLQSYSQADTKQVQMALLDKLVQKW    239
FrpC (Neisseria)      KVELTAEQAKAANLAGIGRLRDLREAAALSGDLANMLKAYSAAETKEAQLALLDNLIHKW    239
FrpA (Neisseria)      KVELTAEQAKAANLAGIGRLRDLREAAALSGDLANMLKAYSAAETKEAQLALLDNLIHKW    239
                       :*** ** :* ** * **:*********  .*:  *: ** *:**: * ***.:*: **

K. negevensis         GATD    242
A. filiformis         AETD    243
FrpC (Neisseria)      AETD    243
FrpA (Neisseria)      AETD    243
                      . **
```

* = fully conserved

: = strongly similar

. = weakly similar

Figure S1: Amino acid alignment of SPM homologs. *Kingella negevensis* SPM is based on the bifunctional hemolysin/adenylate cyclase precursor. *Alysiella filiformis* SPM is based on the hemolysin-type calcium-binding protein related domain-containing protein. FrpC SPM is shown from *N. meningitidis* serogroup B (strain MC58). FrpA SPM is from *N. meningitidis* serotype C.

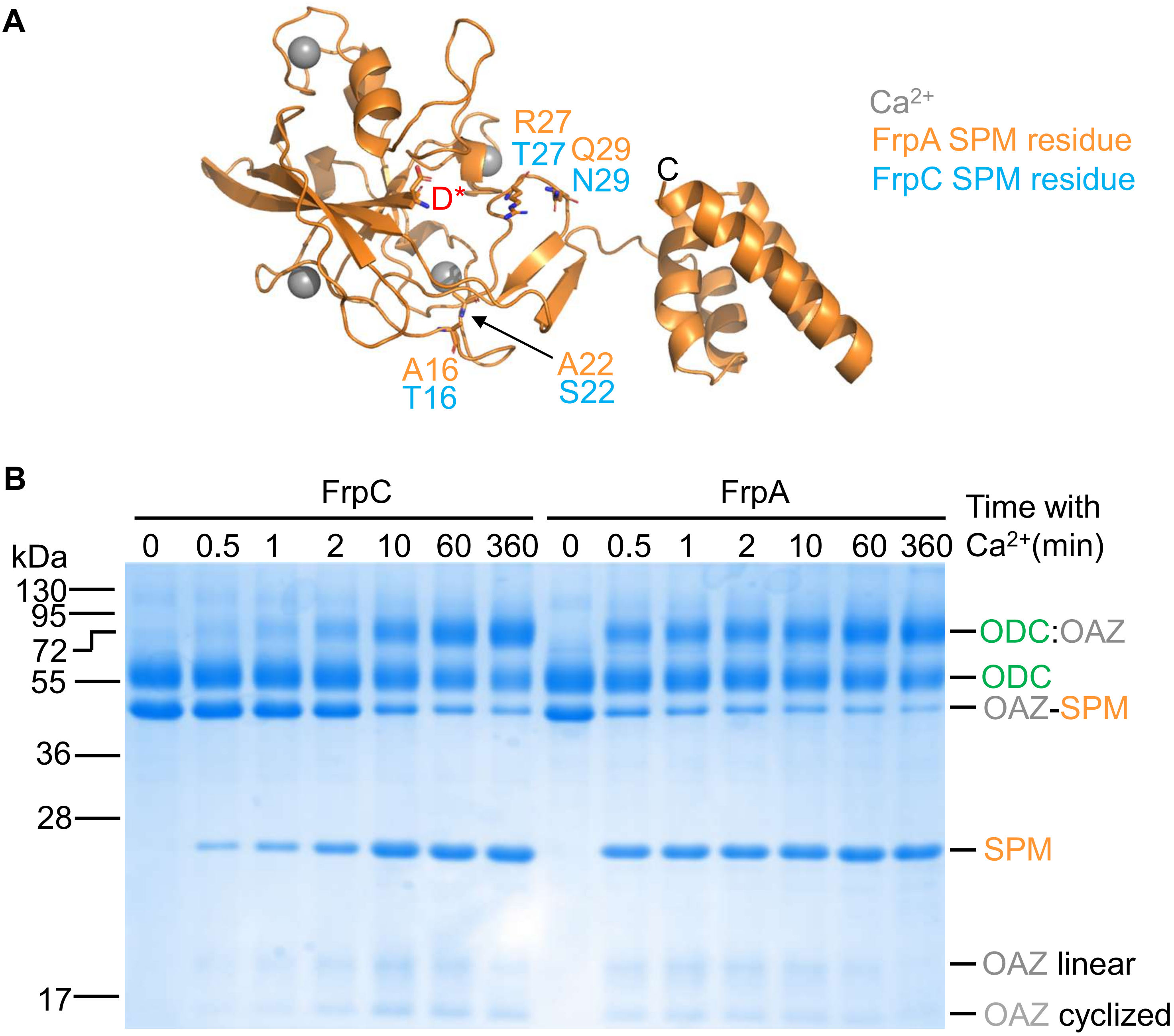

Figure S2: FrpA has faster protein-protein ligation than FrpC. (A) AlphaFold 3 model of FrpA SPM, with calcium as gray spheres. The FrpA residues where there are sequence differences in FrpC are shown in stick format. D* shows the aspartate in stick format where reaction occurs. **(B)** Reaction of 10 μM ODC with OAZ-SPM for FrpC (left) or FrpA (right) for varying times at 25 °C, analyzed by SDS-PAGE/Coomassie.

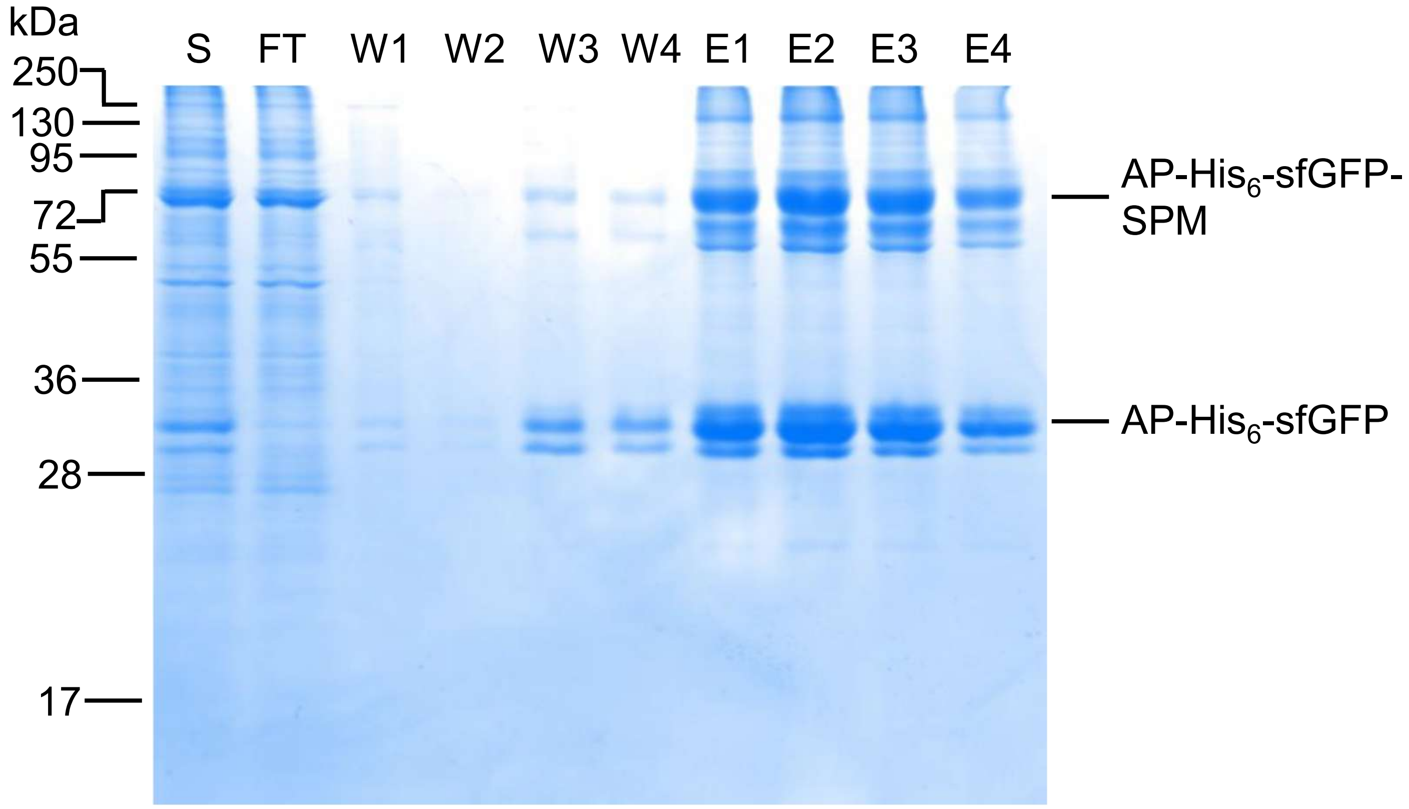

Figure S3: Premature cleavage of AP-His$_6$-sfGFP-SPM after purification from mammalian cells. AP-His$_6$-sfGFP-SPM, targeted for secretion, was purified from the supernatant of Expi293F cells by Ni-NTA after transient transfection. Fractions are analyzed by SDS-PAGE under reducing conditions with Coomassie staining. S supernatant; FT flow-through; W1-W4 column washes; E1-E4 elution fractions. The band corresponding to the molecular weight of full-length AP-His$_6$-sfGFP-SPM is marked, as well as the band corresponding to AP-His$_6$-sfGFP where SPM has been cleaved.

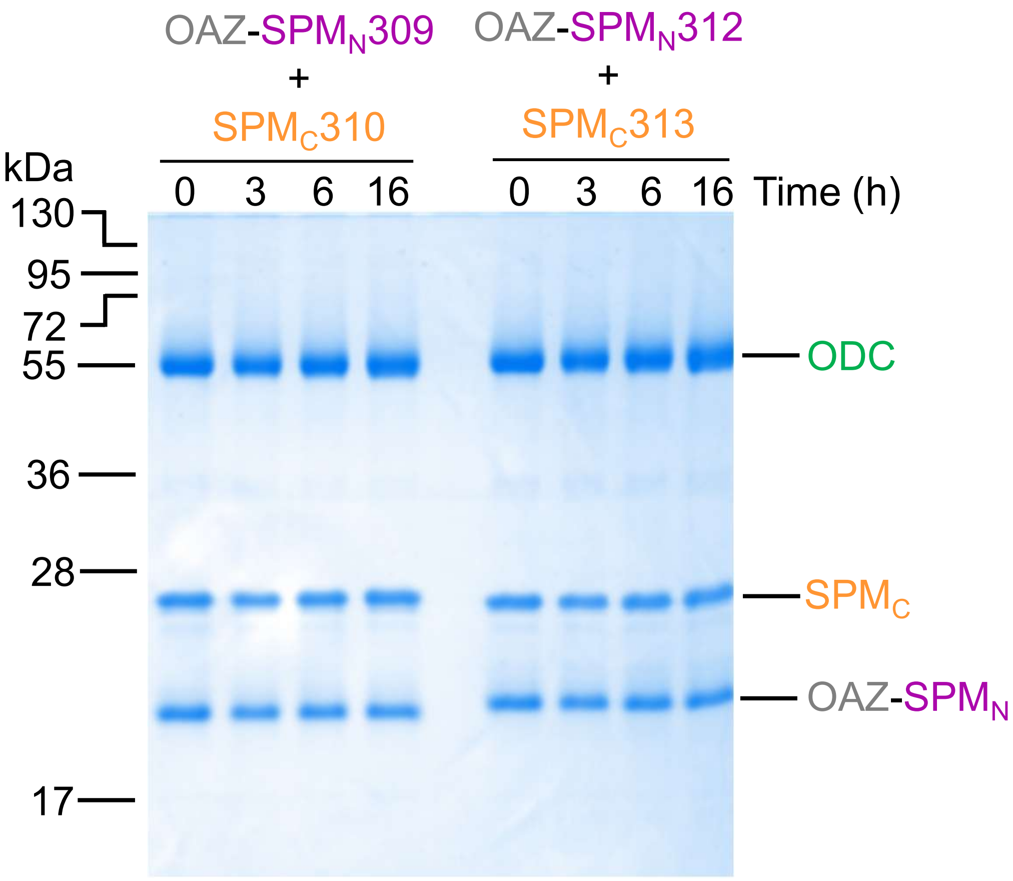

Figure S4: Example N-terminal (OAZ-SPM$_N$) and C-terminal (SPM$_C$) split FrpA constructs that do not reconstitute for reaction. Different 5 μM N-terminal (OAZ-SPM$_N$) and 5 μM C-terminal (SPM$_C$) split FrpA constructs were incubated with 5 μM ODC and activated with 10 mM Ca^{2+} for the indicated time at 37 ^{o}C. No product formation was observed even after 16 h reaction, based on SDS-PAGE with Coomassie staining.

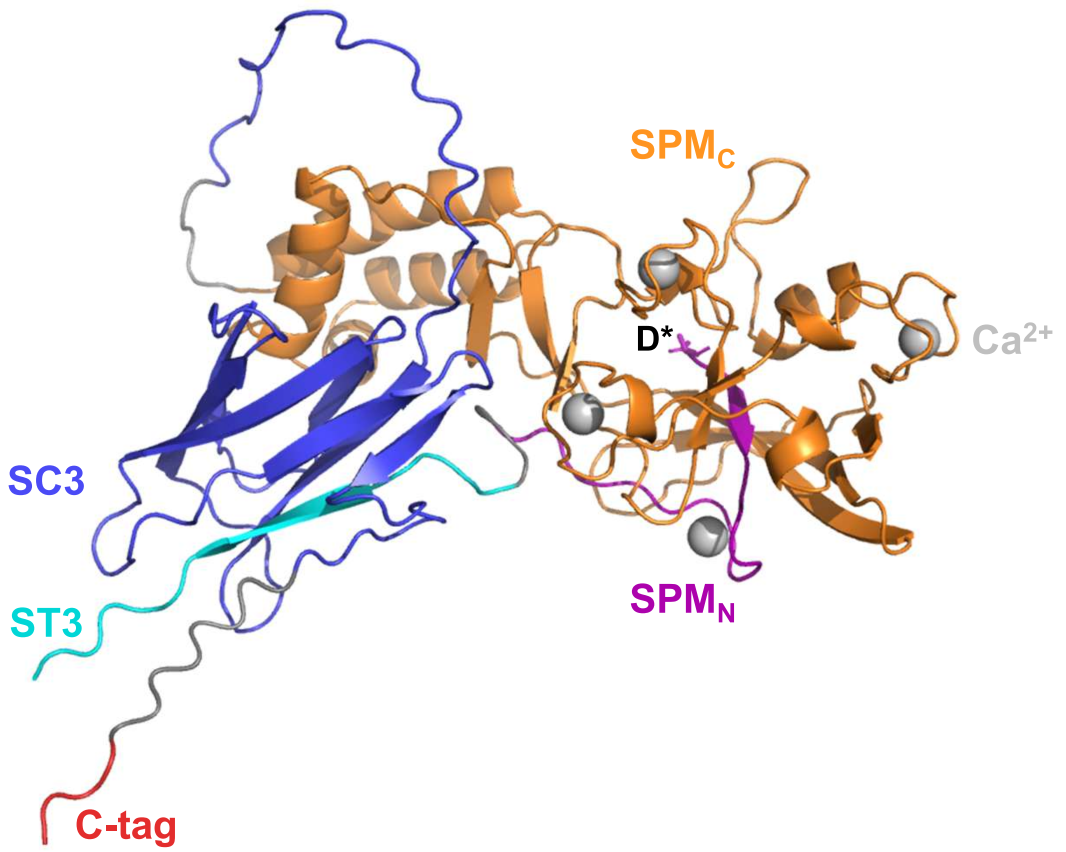

Figure S5: AlphaFold 3 model of the reconstituted SPM_N-ST3:SPM_C-SC3 complex. This model shows the form with calcium ions bound, representing the state before reaction at the aspartate (D*), whose side-chain is shown in stick format. SPM_N in purple; ST3 in cyan; SC3 in dark blue; SPM_C in orange; linkers in gray; C-tag in red; Ca^{2+} as a gray sphere.

Full-length FrpA SPM

```
300 310 320 330 340 350
VYDPLALDLDGDGIETVAAKGFAGALFDHRNQGIRTATGWVSADDGLLVRDLNGNGIIDNG
360 370 380 390 400 410
AELFGDNTKLADGSFAKHGYAALAELDSNGDNIINAADAAFQTLRVWQDLNQDGISQANEL
420 430 440 450 460 470 480
RTLEELGIQSLDLAYKDVNKNLGNGNTLAQQGSYTKTDGTTAKMGDLLLAADNLHSRFKDK
490 500 510 520 530 540
VELTAEQAKAANLAGIGRLRDLREAAALSGDLANMLKAYSAAETKEAQLALLDNLIHKWAE

TD
```

SPM_N-SpyTag003

```
300 310
SYDPLALDLDGDGIETVAAKGGSGRGVPHIVMVDAYKRYK
```

SPM_C-SpyCatcher003-Ctag

```
330 340 350 360 370 380
ALFDHRNQGIRTATGWVSADDGLLVRDLNGNGIIDNGAELFGDNTKLADGSFAKHGYAALA
390 400 410 420 430 440
ELDSNGDNIINAADAAFQTLRVWQDLNQDGISQANELRTLEELGIQSLDLAYKDVNKNLGN
450 460 470 480 490 500
GNTLAQQGSYTKTDGTTAKMGDLLLAADNLHSRFKDKVELTAEQAKAANLAGIGRLRDLRE
510 520 530 540
AAALSGDLANMLKAYSAAETKEAQLALLDNLIHKWAETDGSGAMVTTLSGLSGEQGPSGDM

TTEEDSATHIKFSKRDEDGRELAGATMELRDSSGKTISTWISDGHVKDFYLPGKYTFVET

AAPDGYEVATPIEFTVNEDGQVTVDGEATEGDAHTGSSGSGSGEPEA
```

Figure S6: Amino acid sequences of finalized split NeissLock pair compared to parental FrpA SPM. Residue numbers are based on the full-length SPM. SPM_N in purple; ST3 in cyan; SC3 in dark blue; SPM_C in orange; linkers in black; C-tag in red.

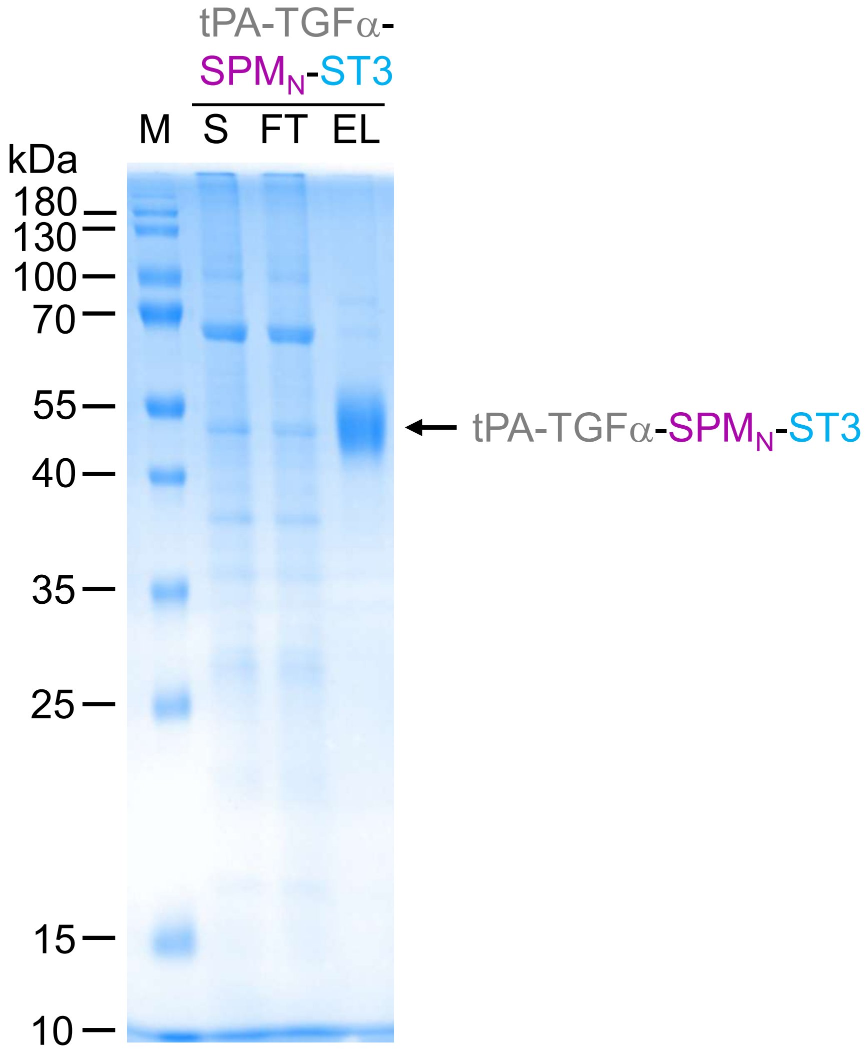

Figure S7: Effective purification of tPA-TGFα-SPM$_N$-ST3 after secretion from mammalian cells. tPA-TGFα-SPM$_N$-ST3 was purified from the supernatant of Expi293F cells by SpySwitch affinity purification after transient transfection. Fractions were reduced with DTT and analyzed by SDS-PAGE with Coomassie staining. M marker; S supernatant; FT flow-through; EL concentrated elution fractions.

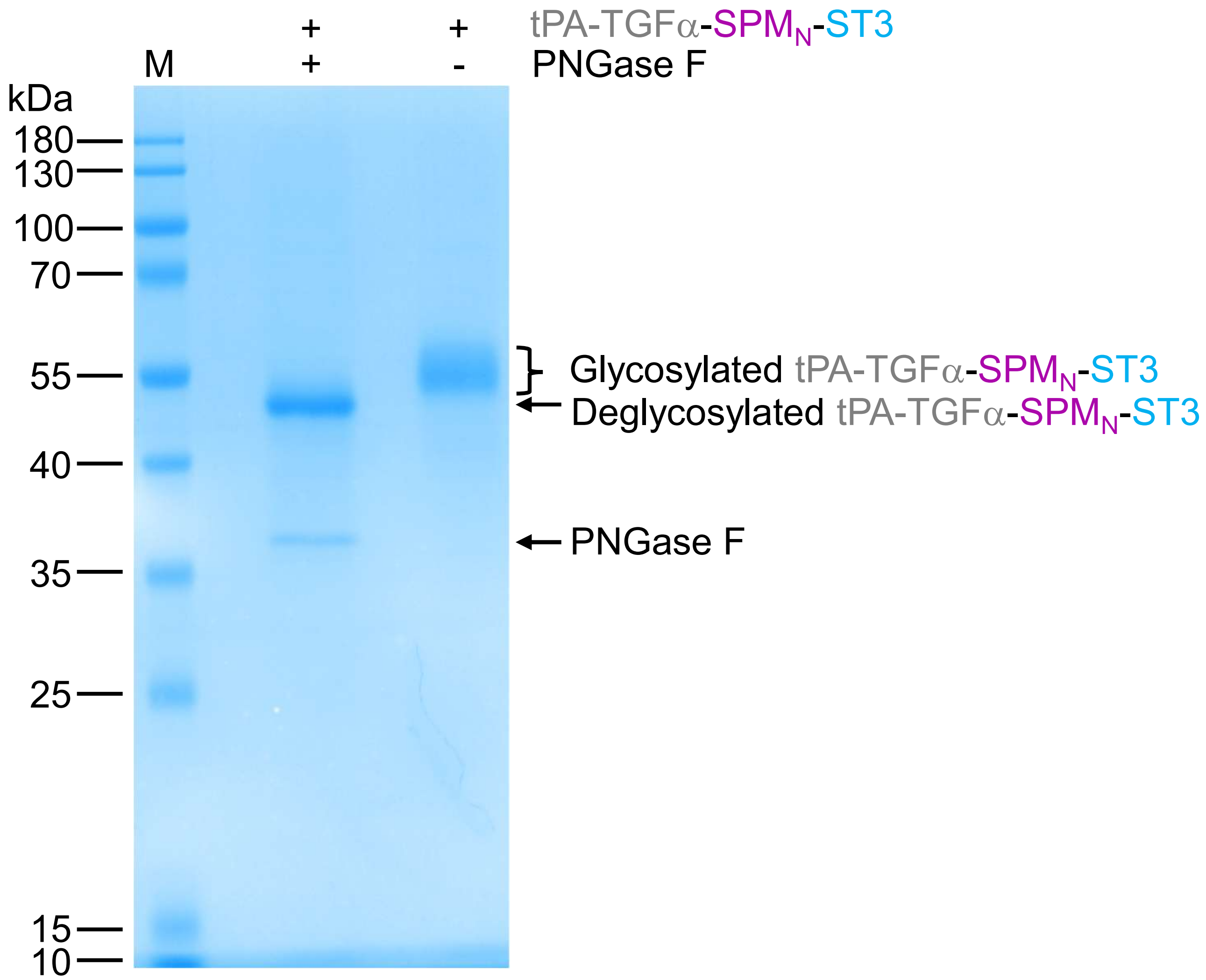

Figure S8: tPA-TGFα-SPM$_N$-ST3 is glycosylated when expressed using Expi293F cells. Purified tPA-TGFα-SPM$_N$-ST3 with or without PNGase F digestion was analyzed by SDS-PAGE with Coomassie staining. M is the molecular weight marker.

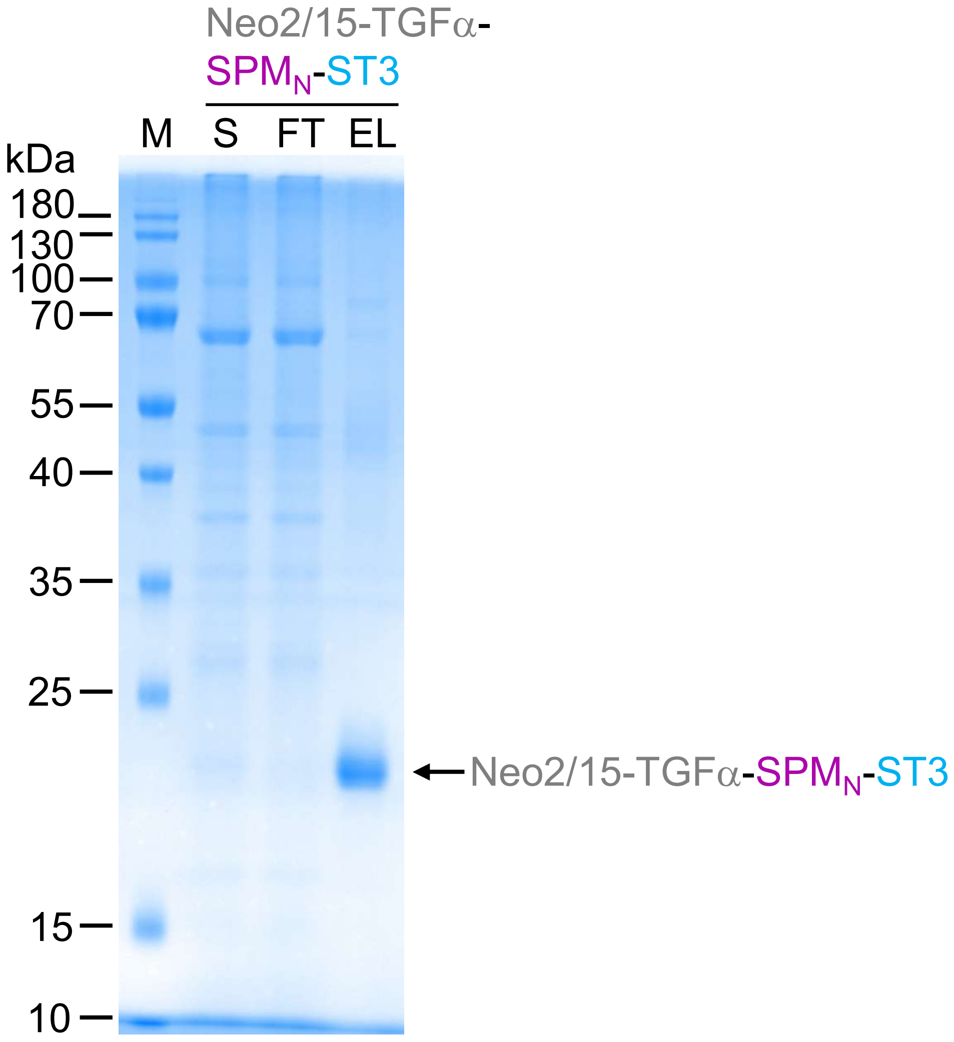

Figure S9: Effective purification of Neo2/15-TGFα-SPM$_N$-ST3 after secretion from mammalian cells. Neo2/15-TGFα-SPM$_N$-ST3 was purified from the supernatant of Expi293F cells by SpySwitch affinity purification after transient transfection. Fractions were reduced with DTT and analyzed by SDS-PAGE with Coomassie staining. M = marker, S = Cell supernatant, FT = column flow-through, EL = concentrated elution fractions

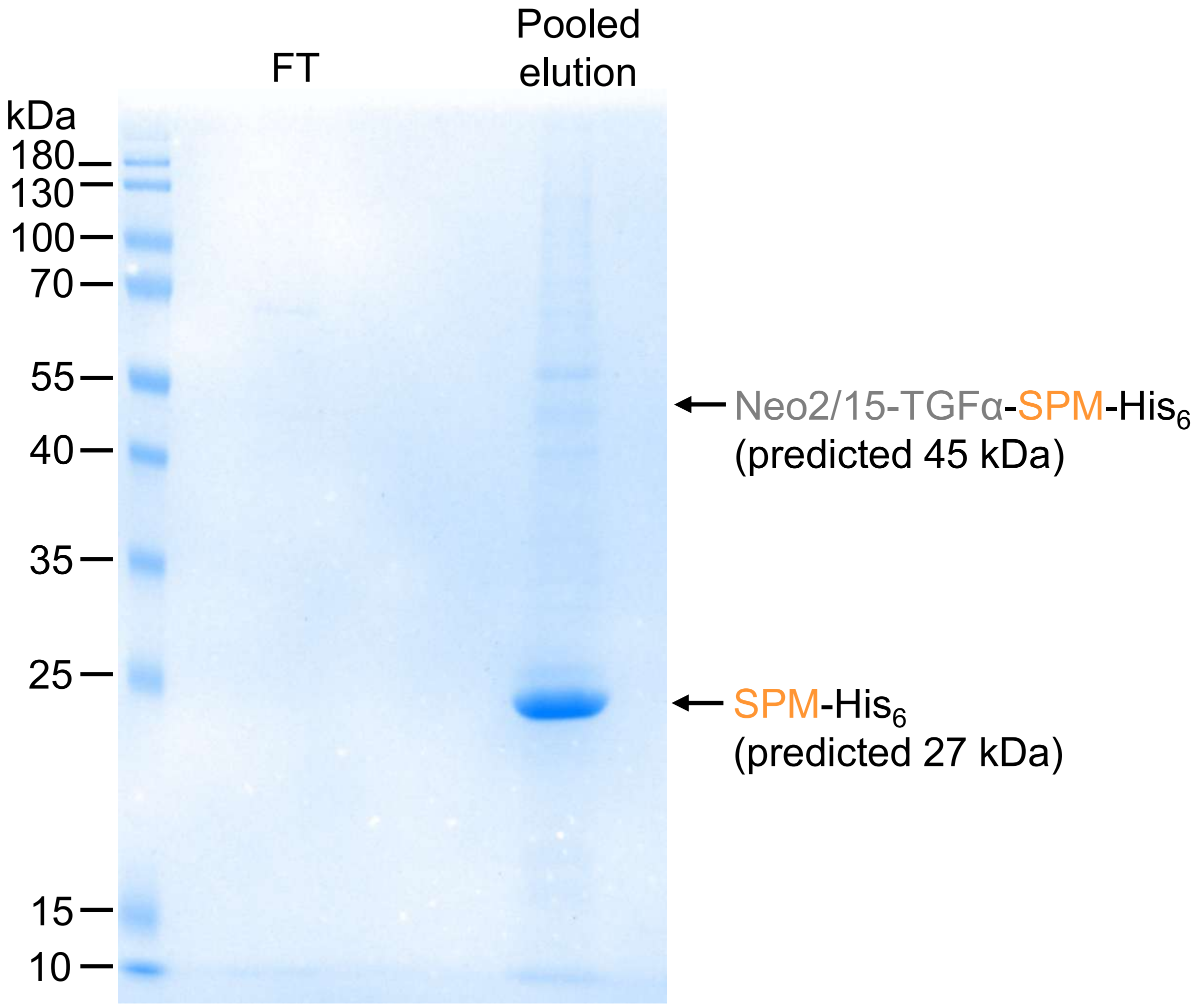

Figure S10: Premature cleavage of Neo2/15-TGFα-SPM-His$_6$ after purification from mammalian cells. Neo2/15-TGFα-SPM-His$_6$ purification from the supernatant of Expi293F cells by nickel-based affinity purification after transient transfection. Fractions are analyzed by SDS-PAGE with Coomassie staining. FT = column flow-through. Minimal full-length Neo2/15-TGFα-SPM-His$_6$ was detected but there was a strong band consistent with the cleaved SPM-His$_6$ product.
